# Metabolite profiling characterises chemotypes of *Musa* diploids and triploids at juvenile and pre-flowering growth stages

**DOI:** 10.1038/s41598-019-41037-z

**Published:** 2019-03-15

**Authors:** Margit Drapal, Elisabete Barros de Carvalho, Mathieu Rouard, Delphine Amah, Julie Sardos, Ines Van den Houwe, Allan Brown, Nicolas Roux, Rony Swennen, Paul D. Fraser

**Affiliations:** 1 School of Biological Sciences, Royal Holloway, University of London, Egham Hill, Egham, Surrey TW20 0EX UK; 2 Bioversity France, Parc Scientifique Agropolis II, 34397 Montpellier, Cedex 5 France; 3 International Institute of Tropical Agriculture, Ibadan, Nigeria; 4 International Institute of Tropical Agriculture, Arusha, Tanzania; 5 Bioversity International, W. De Croylaan 42, 3001 Heverlee, Belgium; 6 Department of Biosystem, KU Leuven University, Oude Markt 13 - bus 5005, 3000 Leuven, Belgium

## Abstract

Bananas (*Musa* spp.) are consumed worldwide as dessert and cooking types. Edible banana varieties are for the most part seedless and sterile and therefore vegetatively propagated. This confers difficulties for breeding approaches against pressing biotic and abiotic threats and for the nutritional enhancement of banana pulp. A panel of banana accessions, representative of the diversity of wild and cultivated bananas, was analysed to assess the range of chemotypes available globally. The focus of this assessment was banana leaves at two growth stages (juvenile and pre-flowering), to see when during the plant growth metabolic differences can be established. The metabolic data corresponded to genomic trends reported in previous studies and demonstrated a link between metabolites/pathways and the genomes of *M. acuminata* and *M. balbisiana*. Furthermore, the vigour and resistance traits of *M. balbisiana* was connected to the phenolic composition and showed differences with the number of B genes in the hybrid accessions. Differences in the juvenile and pre-flowering data led to low correlation between the growth stages for prediction purposes.

## Introduction

The genus *Musa* originated in Southeast Asia and the western Oceania regions and can be divided into several divisions including Eumusa which comprises most edible bananas^1–3^. The two main seeded ancestors for cultivated bananas are *M. acuminata* Colla (A genome) and *M. balbisiana* Colla (B genome). Hybridisation within *M. acuminata* species and between the two genomes led to diploid, triploid and tetraploid cultivars of different genomic composition (e.g. AA, AB, AAB or ABB), that can be identified based on a set of 15 standard morphological descriptors^4,5^. Currently, the production of over 300–400 varieties of banana worldwide is over 144 million tonnes annually and included dessert and cooking bananas. The most commercialised bananas, type Cavendish, account for 47% of the production worldwide^6^.

*M. acuminata and M. balbisiana* have two very different native habitats, namely tropical rainforests in Southeast Asia and monsoon areas in southern Asia, respectively^1,4^, and confer different traits to their progenies. The B genome contributes to a more vigorous growth, whereas the A genome gave rise to parthenocarpy – fruit development without fertilisation – and confers fruit flavour and quality^7,8^. Most banana cultivars are sterile and vegetatively propagated which restricts breeding approaches and most of the natural variation is achieved through spontaneous somatic mutations^7,9,10^. This complicates the generation of segregating population for (i) biotic resistant bananas against e.g. the reoccurring Panama disease^11^ and (ii) more nutritious bananas e.g. higher carotenoid content^12^. On the other side, it emphasises the necessity for banana collections in genebanks e.g. International *Musa* Transit Centre (ITC) and the accumulation of phenotypical information to establish the diversity in present banana accessions e.g. Musa Germplasm Information System (MGIS)^13^. Therefore, the responsibility of genebanks involves conservation of collections and assessment of diversity within these collections which is primarily based on molecular studies^14–18^. These studies in combination with metabolic analysis of the banana plant material can give a better understanding of the value of a variety^19^. In particular, documentation of the chemotype allows elucidation of biomarkers and general mechanisms to facilitate breeding for desired properties^20^. Furthermore, the study of plant metabolism at several growth stages can identify those early growth stages when a chemotype/genotype can be measured to shorten breeding cycles.

In the present study, leaf material of a diversity panel was analysed at the juvenile and pre-flowering growth stage of the banana plant (Table 1). This facilitates the comparison of leaf tissue grown under two conditions (*in vitro* and field) and ascertain the use of *in vitro* grown plants for reduced selection cycles during breeding. Three analytical platforms/approaches were used to cover a broad range of metabolites defining the chemotypes of banana accessions. The chemotypes observed differed according to genomic composition and gave an overview of the metabolic diversity in the present *Musa* panel.

**Table 1:** List of *Musa* varieties included in the study.

Accession number	Accession name	Subspecies/Subgroup	Genome Group	Consumption type of fruit^52,53^	Leaf stage
ITC0084^1^	Mbwazirume	Mutika/Lujugira	AAA	cooking	JP PF
ITC0111^1^	Agbagba	Plantain	AAB	cooking	JP PF
ITC0112^1^	Bobby Tannap	Plantain	AAB	cooking	JP PF
ITC0121^1^	Ihitisim	Plantain	AAB	cooking	JP PF
ITC0123^1^	Simili Radjah	Peyan	ABB	cooking	PF
ITC0180^1^	Grande Naine	Cavendish	AAA	dessert	JP
ITC0245^1^	Safet Velchi	Ney Poovan	AB cv	dessert	JP PF
ITC0249^1^	Calcutta 4	*burmannica*	AA w	n/a	JP PF
ITC0250^1^	Malaccensis Holotype	*malaccensis*	AA w	n/a	JP PF
ITC0253^1^	Borneo	*microcarpa*	AA w	n/a	JP PF
ITC0277^1^	Leite	Rio	AAA	dessert	PF
ITC0283^1^	Long Tavoy	*burmannica*	AA w	n/a	JP
ITC0312^1^	Pisang Jari Buaya	Pisang Jari Buaya	AA cv	dessert	JP PF
ITC0361^1^	Blue Java	Ney Mannan	ABB	both	PF
ITC0472^1^	Pelipita	Pelipita	ABB	cooking	PF
ITC0587^1^	Pisang Raja (South Johnstone)	Pisang Raja	AAB	both	PF
ITC0609^1^	Pahang	*malaccencis*	AA w	n/a	JP
ITC0623^1^	Banksii	*banksii*	AA w	n/a	JP
ITC0643^1^	Cachaco	Bluggoe	ABB	cooking	JP
ITC0649^1^	Foconah	Pome	AAB	dessert	PF
ITC0653^1^	Pisang Mas	Sucrier/Figue	AA cv	dessert	JP PF
ITC0654^1^	Petite Naine	Cavendish	AAA	dessert	JP PF
ITC0659^1^	Namwa Khom	Pisang Awak	ABB	dessert	JP PF
ITC0662^1^	Khai Thong Ruang	Ibota	AAA	dessert	PF
ITC0767^1^	Dole	Bluggoe	ABB	cooking	PF
ITC0769^1^	Figue Pomme Géante	Silk	AAB	dessert	PF
ITC1120^1^	Tani	*balbisiana*	BB	n/a	PF
ITC1121^1^	Pisang Lilin	Pisang Lilin	AA cv	dessert	PF
ITC1122^1^	Gros Michel	Gros Michel	AAA	dessert	PF
ITC1177^1^	Zebrina	*zebrina*	AA w	n/a	JP PF
ITC1187^1^	Tomolo	AA	AA cv	Cooking	JP PF
ITC1325^1^	Orishele	Plantain	AAB	cooking	JP PF
ITC1330^2^	*Musa ornata*	*ornata*		n/a	JP
ITC1441^1^	Pisang Ceylan	Mysore	AAB	dessert	PF
ITC1483^1^	Monthan	Monthan	ABB	cooking	PF
ITC1511^1^	CIRAD 930	*malaccencis*	AA w	n/a	JP
ITC1527^1^	*Musa balbisiana*	*balbisiana*	BB	n/a	JP
ITC1587^1^	Pisang Klutuk Wulung	*balbisiana*	BB	n/a	JP

Accessions are listed with number/name, classification (Eumusa^1^ and Rhodochlamys^2^), subspecies, genome group (w, wild; cv, cultivars), consumption type of the fruit (n/a, not applicable) and which leaf stage was analysed (JP, juvenile plantlets; PF, pre-flowering leaves).

## Results and Discussion

**Figure 1:**
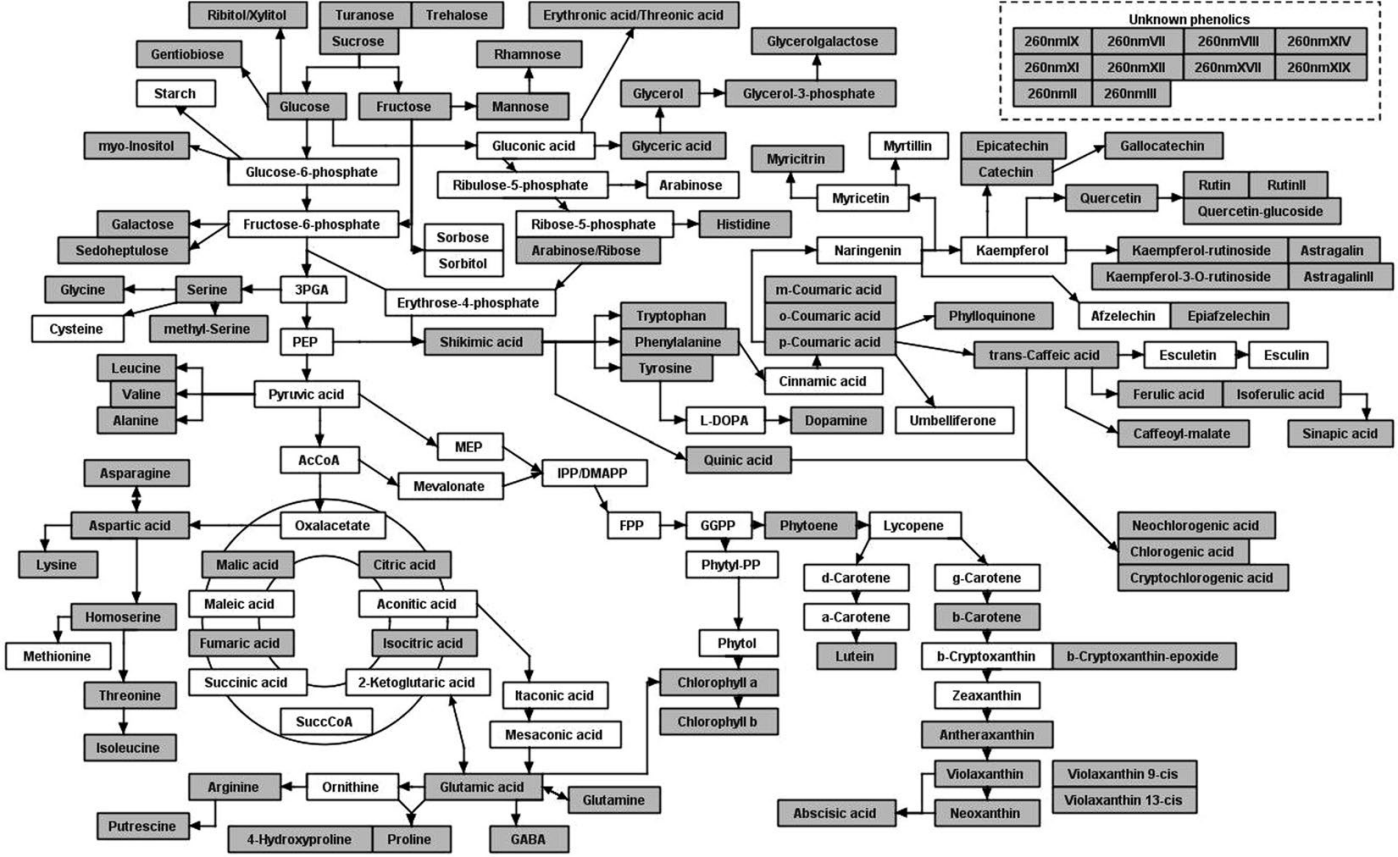
Pathway display of metabolites detected for juvenile plantlets and pre-flowering leaves. All metabolites specific to *Musa spp* identified through LC-MS, GC-MS and UPLC-DAD were highlighted (grey) in the pathway display constructed with BioSynLab software^©^ (RHUL, UK).

The biochemical composition of a *Musa* spp diversity panel (38 accessions) was assessed by screening polar and non-polar extracts of juvenile plantlets and pre-flowering leaves on three different platforms to gain a comprehensive view of the metabolome^21^. The diversity panel (Table 1) included diploid and triploid genome groups of the section Eumusa: specimen of the two wild species *M. acuminata* and *M. balbisiana*, cultivated AA (AAcv), AAA, AAB, AB, ABB and one *M. ornata* accession of the section Rhodochlamys. The metabolites identified (~105, Supplementary Table S1) comprised a range of intermediates of primary and secondary metabolic pathways (Fig. 1). Relative quantification was carried out and combined into one data set (Supplementary Tables S2 and S3).

### Diversity panel – juvenile leaves

Twenty-four accessions were analysed at the juvenile growth stage of the plant (Table 1). The similarities between accessions and genome groups based on the metabolite composition will be discussed in comparison to several genotyping studies, each comprising a majority of the accessions at a given time.

**Figure 2:**
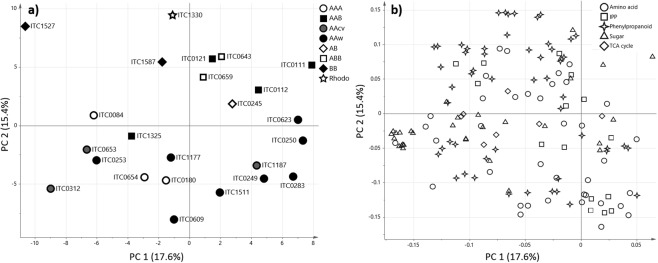
PCA analysis of juvenile plantlets. PCA score plot (**a**) included all accessions analysed. PCA loadings plot (**b**) included all metabolites identified in the targeted analysis. Abbreviations: Rhodo, Rhodochlamys; AAw, wild *M. acuminata*; AAcv, cultivated AA; IPP, isopentenyl pyrophosphate derived pigments.

**Figure 3:**
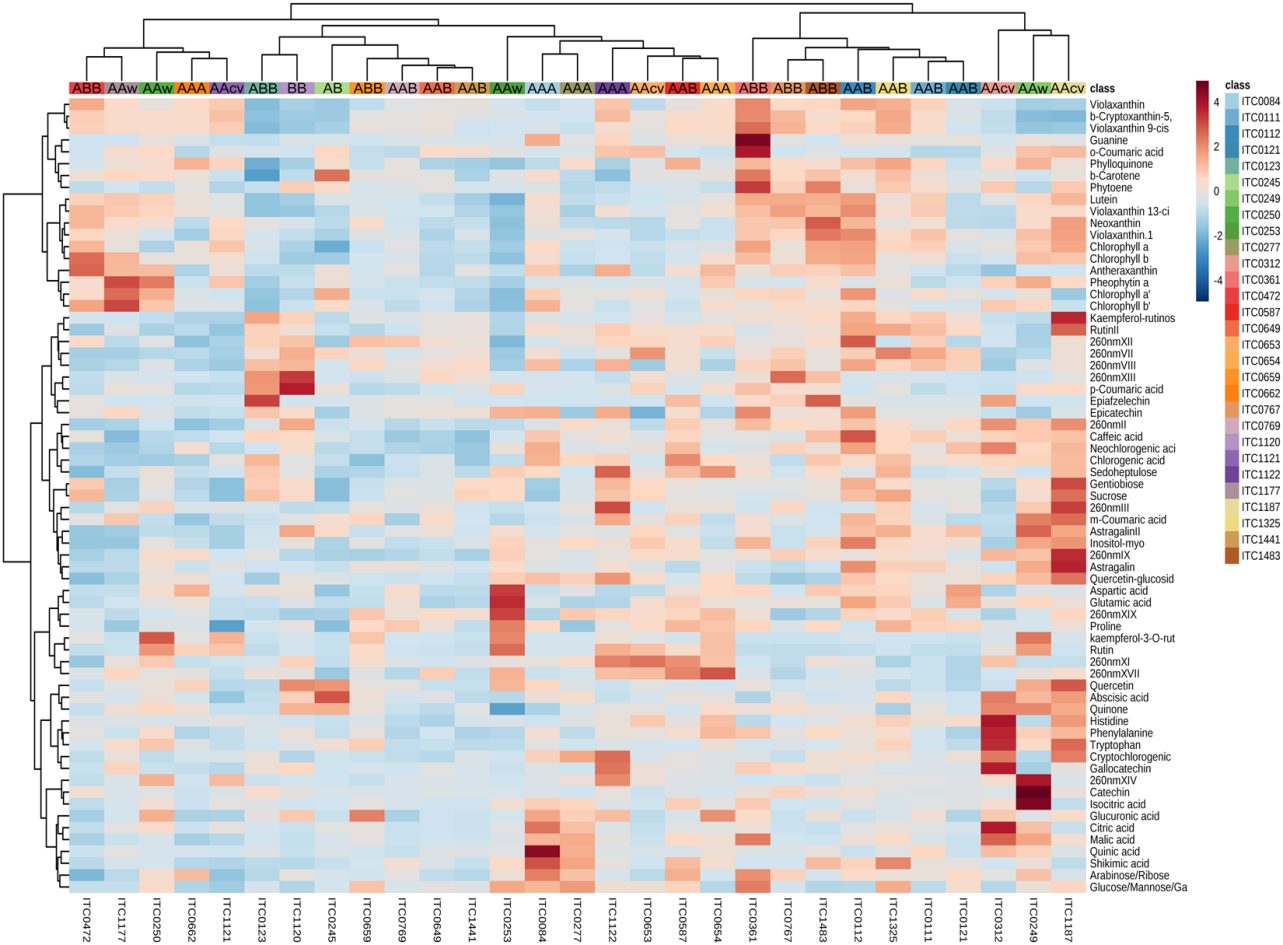
Heatmap of juvenile plantlets. Genotypes were grouped based on the similarity of their metabolite composition. Genome group of the accessions is displayed in the heatmap.

Principal component analysis (PCA) of the metabolite data showed that the widest metabolic diversity was primarily found in the wild *M. acuminata* and *M. balbisiana* accessions (Fig. 2a). This is consistent with genetic diversity studies showing the majority of cytoplastic patterns were found in the wild *M. acuminata* accessions^14,22^. Another general trend of the metabolite data was a distinct separation between accessions containing only the A genome and at least one B genome, further referred to as group A and group B (Fig. 2a). *M. ornata*, the only accession of the section Rhodochlamys, clustered on the far side of group B. Genetic studies are in contradiction whether *M. ornata* is more similar to *M. balbisiana*^15,23,24^ or *M. acuminata*^16,17^, but the metabolite profile of this accession suggests a chemotype more similar to the B genome. The wild *M. acuminata* accessions showed a mixed cluster of *malaccensis* and *burmannica* subspecies, which were located closer to the *banksii* accession than the *zebrina* and *microcarpa* accessions. This PCA pattern was also detected based on genotyping data, identifying *zebrina* as the most different of the wild *M. acuminata*^25^. Additionally, the metabolite composition of wild *M. acuminata* species is a representation of their geographical locations^3,26^ and of the heterozygosity in *M*. *acuminata* species as displayed by the *malaccensis* accessions^8^. The exception was the *banksii* accession (ITC0623), which was located close to Plantains (AAB) and correlates with the strong presence of *banksii* genes in the putative progeny of Plantains^2,26^. No definitive separation was observed between the genome groups wild *M. acuminata*, AAcv and AAA, which might be a result of backcrossing and the dominance of maternal and paternal genes^23,27^. A comparison of wild and cultivated accessions of *banksii, microcarpa and malaccensis* showed no common change of metabolite features between the wild and cultivated states (Supplementary Fig. S4). The PCA score plot indicated a different metabolite composition of Tomolo (ITC1187, AAcv) compared to the other two AA cultivars Psiang Jari Buaya (ITC0312) and Psiang Mas (ITC0653), which was visualised in the heatmap (Fig. 3) and coincides with DNA profiling^15^ and the difference in maternal parents^14,22^. A similar case was found for Mbwazirume (ITC0084) an East African Highland banana of *banksii* and *zebrina* origin, which displayed a different chemotype from other two AAA accessions, related to all other subspecies^14,22,28^. Additionally, Mbwazirume was located in the upper half of the score plot with group B, indicating that farmers’ selection for a starchy pulp phenotype or the adaptation to a geographical location outside the natural range of the species led to a metabolic phenotype more similar to plantain cultivated in Africa (Figs 2 and 3). The close grouping of the other two AAA Petite naine (ITC0654) and Grande naine (ITC0180), both Cavendish accessions, confirms that an identical genomic background leads to a very similar metabolic composition^14^. The only diploid hybrid composed of A and B genome, Safet Velchi (ITC0245), was located between the groups A and B and closer to accessions of the genome groups AAB and ABB than wild *M. balbisiana*^17^. This indicates a strong influence of the B genome on the metabolome, as Safet Velchi was located with group B despite the prominent genetic background of *malaccensis*^14,15^.

As observed for group A, the accessions of genomic compositions AAB and ABB did not have enough metabolic differences to separate on the score plot. Previous studies showed the close genetic relation between AAB and ABB as well as the different cytoplasmic DNA patterns leading to the observed chemotypes^8,14,18^. Interestingly, Orishele (ITC1325) was the only Plantain that clustered in the lower half with group A. The different metabolite composition of Orishele and some genetic studies indicate a change in this particular variety compared other Plantains, which are known for their genetic similarities^22,29,30^. The genetic study by Ude, *et al*.^29^ described Agbagba (ITC0111) as the most different Plantain variety as seen from the location of Agbagba in the PCA towards the edge of the plot (Fig. 2a). Within the *balbisiana* accessions, Pisang Klutuk Wulung (ITC1587, origin Indonesia) clustered towards AAB and ABB accessions, whereas *M. balbisiana* (ITC1527, origin China) was clustered away the furthest from all accessions analysed. *M. balbisiana* plants originate in continental Asia and are now widely distributed throughout the tropical and subtropical regions of Asia^31^. Analysis of cytoplasmic DNA confirmed several patterns unique to the B genome^22,23,32,33^, which could lead to very different chemotypes as reflected in the PCA analysis of metabolite data. Additionally, the similar metabolite composition between Pisang Klutuk Wulung (ITC1587) and triploids containing a B genome indicates the participation of Pisang Klutuk Wulung or a similar BB accession in the hybridisation of subgroups in the East contact area^31^. This contradicts Li, *et al*.^22^, who concluded that neither Pisang Klutuk Wulung nor Tani (ITC1120, pre-flowering data set) were involved in domestication of cultivated bananas due to the presence of *Waxy* gene. The similar metabolite composition of the two *M. balbisiana* accessions to triploids of group B suggests that the presents of some genes might not influence the primary metabolism (Figs 2 and 3). Furthermore, the results emphasise the need for a more detailed study of *M. balbisiana* accessions throughout tropical and subtropical Asia as the studies from specific regions^23,32–34^ do not provided enough information about the actual diversity of the B genome.

Differential expression of the metabolites detected showed that ~37% of the metabolites were significant to distinguish group A and B. These metabolites include isopentenyl pyrophosphate derived pigments such as chlorophylls and carotenoids (IPPs), dopamine, amino acid precursors for phenolics and for fatty acids with a higher mean in group A and amino acids involved in the nitrogen cycle, chlorogenic acids and catechins with a higher mean in group B (Supplementary Table S5). This comparison emphasises the improved vigour and tolerance to stresses associated with the B genome conferred partly by a higher content of phenolics^35–37^. A more detailed differential expression between genomic compositions showed ~75% of metabolites were significantly different. These metabolites comprised all chemical classes detected in the dataset (Supplementary Table S5). The two major chemical classes differentiating group A and B were IPP derived pigments and phenylpropanoids. The levels of most IPPs were higher in group A than group B which indicates differences in the regulation of photosynthetic processes and in the inheritance of photosynthesis related genes in interspecific hybrids. Genes encoding proteins involved in photosynthesis are located in the chloroplastic DNA (cpDNA) as well as the nucleus^38^. Changes in the regulatory network of photosynthetic processes can overcome climatic challenges^39,40^. Hence, the differences in the production of photosynthesis related antioxidants is to be expected between *M. acuminata*, which is prevalent in warmer regions with higher levels of precipitation, *and M. balbisiana*, cultivated in dryer, moderate warm regions^14,41–43^. Interestingly, the levels of IPPs in AAB were higher than wild *M. balbisiana*, whereas ABB and AB had up to 20% lower levels of IPPs compared to wild *M. balbisiana* which suggests that the inherited chloroplastic genome has a prominent role in the composition of photosynthetic metabolites^26,27^. The detected phenolics participate in structural and chemical barriers against abiotic and biotic stresses. Phenolic acids (e.g. caffeic and coumaric acid) and flavonols (e.g. kaempferol, quercetin and their glycosides) act as phytoanticipins, compounds synthesised before an attack, whereas catechins and chlorogenic acids are involved in the lignin biosynthesis, a physical barrier^44,45^. The highest levels of phenolics, involved in both structural and chemical barriers, were detected in wild *M. balbisiana* accessions. Interestingly, chlorogenic acid and dopamine (a tyrosine derived phenol) were detected in wild *M. acuminata* with similar levels to wild *M. balbisiana*, which suggests fundamental resistance mechanism are present in the wild *M. acuminata* species^44,46^. The *burmannica* accession Calcutta 4 (ITC0249) is well known for its resistance against several biotic stresses and yet the precise resistance mechanism have not been identified^47,48^. The metabolite data of Calcutta 4 showed average overall phenolic levels and above average levels of rutin, chlorogenic acid and caffeoyl-malate. This would suggest the resistance traits of Calcutta 4 is based on the regulatory processes engaged during/after the infection. The fact that the metabolite data showed the highest levels of phenolics precursors (phenylalanine and tyrosine) in group A, indicates that the activity of phenylalanine ammonia lyase (PAL), the committed and rate-limiting enzyme in the phenolics pathway, is regulated differently in the A and B genome. Additionally, the presence of the less efficient PAL from the A genome seems to affect accessions containing both genomes. Accessions from AAB, ABB and AB genome groups had significantly lower levels than wild *M. balbisiana*, which were nevertheless significantly higher than AAA and AAcv.

### Diversity panel – Pre-flowering leaves

**Figure 4:**
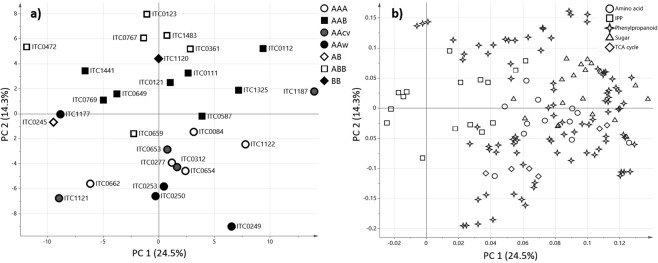
PCA analysis of leaves of pre-flowering plants. PCA score plot (**a**) included all accessions analysed. PCA loadings plot (**b**) included all metabolites identified in the targeted analysis. Abbreviations: AAw, wild *M. acuminata*; AAcv, cultivated AA; IPP, isopentenyl pyrophosphate derived pigments.

**Figure 5:**
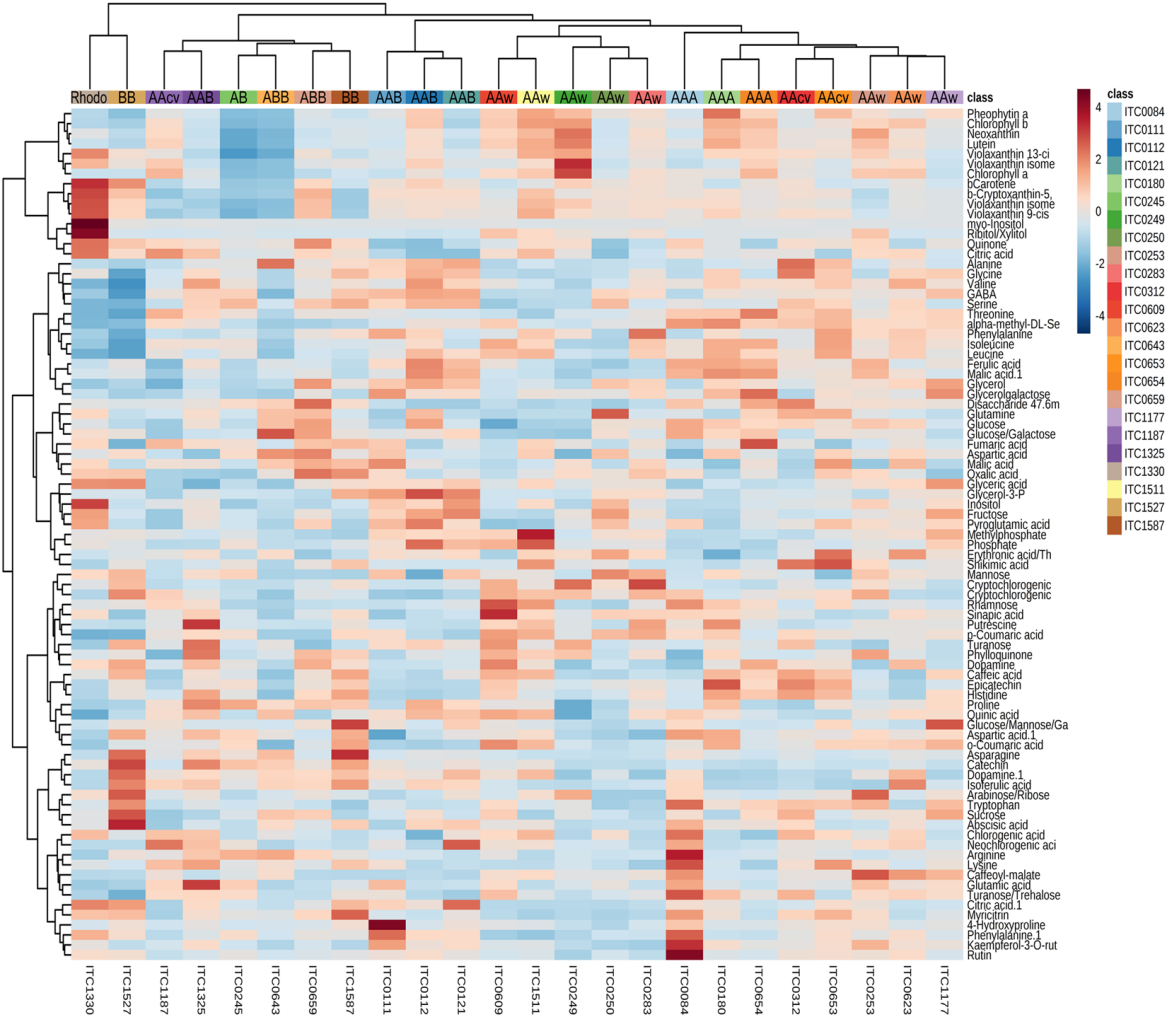
Heatmap of preflowering plants. Genotypes were grouped based on the similarity of their metabolite composition. Genome group of the accessions is displayed in the heatmap.

The accessions analysed at the pre-flowering growth stage of the plant included 29 varieties comprising all genome groups except the Rhodochlamys accession *M. ornata* (Table 1). PCA analysis of the leaf metabolite profile showed a separation of group A and B along the horizontal axis (Fig. 4), as observed for the data at the juvenile stage. Many other properties of the PCA plot of pre-flowering leaf were similar to the juvenile leaf data and included the location of the AB accession between group A and B, no separation between the genome groups AAA, wild *M. acuminata* and AAcv and the location of *zebrina* away from other wild *M. acuminata*. Despite lack of separation between A groups, the majority of AAA seemed to cluster around two AAcv Pisang Jari Buaya (ITC0312) and Pisang Mas (ITC0653), which might suggest those accessions emerged from the same genepool^22,49^. A significant difference between the juvenile and pre-flowering stages was the clear separation of AAB and ABB accessions into two cluster, as described by several genotyping studies^18,24,50^. The wild *M. balbisiana* Tani (ITC1120) was located between these two genome groups as described for the juvenile data set. Within the ABB accessions, Pelipita (ITC0472) had the lowest allelic contribution of *banksii*^26^ and was located away from the other ABB accessions towards the left edge of the score plot. A similar trend could be seen for Pisang Rajah (ITC0587) an AAB accession which was located in the middle of the score plot towards group A. Another difference between the growth stages was that the *microcarpa* accession was more similar to the *malaccensis* than the *zebrina* accession (Fig. 4). This metabolite data matches the chemotypes expected from mitotype^14^ and geographical origin of *microcarpa and malaccensis*^1,2^. Additionally, the pre-flowering leaves showed a metabolic trend in the PCA plot based on consumption type. This was visualised with several group B accessions located towards wild *M. acuminata* and cooking pulp types of group A towards group B accessions with the same pulp type. Specifically, Tomolo (ITC1187,AAcv) was located in the upper half whereas Namwa Khom (ITC0659, ABB) was located in the lower half of the score plot (Fig. 4). The metabolite profile of Namwa Khom is of special interest as it has the same cytoplasmic background as Simili Radjah (ITC0123)^14,26,27^, which was clustered towards to top edge of the score plot. Nevertheless, the breeding schemes for these two ABB accessions must have led to different chromosome re-assortment during the exchange of chromosome segments, which resulted in very different regulatory processes for the metabolome^27,51^. This can also be seen in the metabolite composition (Fig. 5). Furthermore, the remaining genes from the A progenitor in Namwa Khom could be responsible for a metabolic composition of the pulp that is preferably eaten as a dessert banana contrary to other ABB varieties such as Simili Radjah (Table 1)^52,53^. Previous studies evidenced that the metabolism of the leaf (i) changes slightly before emergence of flowers^54,55^ and (ii) is connected to the sink organ^56,57^. In the latter studies, the PCA of leaf and tuber/root showed the same metabolic trends. Furthermore, the metabolite data from the present study emphasises the finding that cooking characteristics are not related to the B genome^58^.

Differential expression showed that ~27% of the metabolites – less than juvenile data – were significantly different between group A and B and included primarily phenylpropanoids (Supplementary Table S6). Glycosylated phenylpropanoids were in general higher in group A, contrary to the juvenile data, and catechin levels were higher in group B as observed for the juvenile leaves. No dopamine was detected in the leaves at pre-flowering stage. The differential expression between genome groups showed that only ~52% of metabolites were significantly different and mainly comprised phenolics and IPPs. In the pre-flowering leaves higher levels of glycosylated phenolics were detected in group A which might be related to storage in the vacuole rather than transport to further synthesis locations^59^. Epiafzelechin, a flavonoid monomer unit for tannins^60^, and the majority of phenolics had the highest means in ABB and wild *M. balbisiana* accessions. The amount of free phenolics in the ABB accessions indicates a more effective resistance as well as cell wall strengthening through lignification compared to AAB accessions^8,60,61^. This hypothesis needs confirmation with environmental studies involving biotic and abiotic stresses similar to the effects of Fusarium wilt on banana roots^62^. Interestingly, accessions of genome groups wild *M. acuminata* and AAcv had similar levels of phenolics compared to AAB. This data implies dramatic changes in the metabolic regulation from the juvenile to the pre-flowering growth stage, probably related to preparations for flowering as mentioned earlier^54,55^.

### Correlation of juvenile and pre-flowering leaves

**Figure 6:**
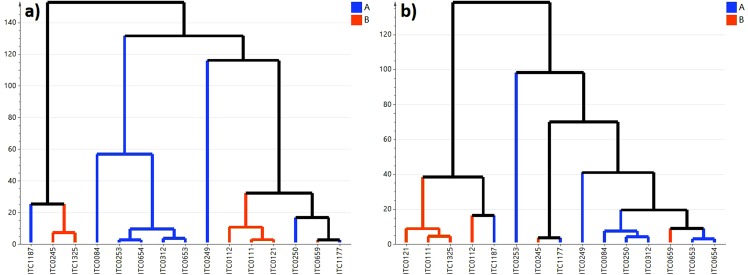
Hierarchical clustering analysis of juvenile and pre-flowering leaves. Agglomerative hierarchical clustering (AHC) was performed for individual accessions (**a**,**b**) at the juvenile (**a**) and pre-flowering (**b**) growth stage. Clustering was based on averaged metabolite data detected in accessions present in both growth stages. The AHC of accessions was coloured by accessions contained in group A (blue) and group B (orange).

The diversity panels studied at juvenile and pre-flowering growth stages included 15 accessions present in both panels (Table 1). Metabolite composition at both stages were compared to assess the potential of predicting the pre-flowering chemotype from the juvenile plantlet. Two comparisons were conducted to evaluate prediction based on the genome group and on the specific accessions (Fig. 6). The dendrogram based on accessions was similar between juvenile and pre-flowering growth stages in a few aspects, such as grouping of Plantains (Agbagba, Bobby Tannap and Ihitisim) (Fig. 6a,b). Both the RV coefficient (*R* = 0.784, *p* = 0.016) and Mantel test were significant (*p* = 0.02) and ascertain that similar metabolite trends can be observed in the leaves of juvenile and pre-flowering plants. Multifactor analysis combing the PCA plots of the two growth stages (*R* = 0.577) highlighted that Zebrina, Pisang Jari Buaya, Safet Velchi, Calcutta 4 and Mbwazirume showed the most difference between juvenile and pre-flowering stage causing the low correlation between the growth stages. Based on this data, the correlation between group A and B was significant between the growth stages based on Mantel test (*p* = 0.008) and RV coefficient (*R* = 0.53, *p* = 0.007). The association tests of data summarised by genome groups showed no significant correlation between the juvenile and pre-flowering data sets (RV coefficient: *R* = 0.288, *p* = 0.459 and Mantel test *p* = 0.731).

The present study provides evidence to suggest that the metabolic changes occurring over the two growth stages show a low association on the level of accessions and group A/B and predictions from juvenile plantlets to fully grown plants should be performed with precaution. Several factors could be responsible for the low correlation between the two growth stages. Firstly, the leaves of juvenile plantlets were obtained from tissue culture grown plants. The leaves at this stage are small and hence, during sampling the leaf part of the petiole or even the leaf sheath might have been included. The latter two leaf parts are transport organs with very transient metabolism^63^ (Drapal *et al*., submitted) and can affected the overall composition of the juvenile leaves analysed. Secondly, the leaves undergo anatomical and morphological changes during acclimatisation from *in vitro* to the field^64^. Lastly, epigenetic changes are known to occur during *in vitro* propagation on multiple levels and can affect the plant’s regulatory networks (e.g. genome and metabolome) and morphology as reviewed by Us-Camas, *et al*.^65^. If the epigenetic effects were temporary, as previously reported, then the metabolism of the *in vitro* and field grown material will show differences. Hence, to ascertain the usefulness of *in vitro* plants for prediction in the field a study with a wider variety of banana accessions including genotyping and metabolomics concurrently would be necessary.

In conclusion, the present study revealed that the complex genetic backgrounds of banana accessions have a specific metabolite phenotype. The accession specific chemotypes differ between *in vitro* and field grown plants, which bears challenges for accelerating breeding programs by screening young plantlets. Nevertheless, both cultivation conditions showed a clear separation between accessions containing solely the A genome and at least one B genome. Furthermore, the metabolite profiles indicated that the combination of *M. acuminata* and *M. balbisiana* genome in hybrids can interfere with certain pathways e.g. photosynthetic metabolites and phenolics biosynthesis. As previously reported, the resistance traits associated with the B genome is mediated by phenolic compounds. More specifically, a prominent difference between the two genomes is the ubiquitous presence of elevated phenolics involved in structural and chemical defence in the B genome. Future metabolite analysis of pulp and peel will show whether these metabolite differences can also be detected in the consumed banana product(s) and influence the banana pulp quality.

## Material and Methods

### Plant material

Banana leaf samples were supplied by Bioversity International *Musa* Transit Centre (ITC), hosted at KU Leuven (Belgium), and International Institute of Tropical Agriculture (IITA) in Ibadan, Nigeria. The banana accessions analysed comprised diploid and triploid, wild and cultivated accessions (Table 1). The plants obtained from ITC were *in vitro* propagated plants harvest and present juvenile banana plantlets (JP). The field material obtained from IITA was grown in Ibadan, Nigeria research farm. Field material (third open leaf) was harvest from 6-month old plants, which presents the pre-flowering (PF) growth stage of banana plants. All leaf material was immediately frozen in liquid nitrogen and lyophilised.

### Metabolite extraction

Dried tissue was ground up and pooled quality controls (QC) prepared separately for juvenile and pre-flowering leaf sets. Extraction of metabolites followed previously published protocols and included randomisation of samples and extraction in sets of 20 samples with one QC and one extraction blank^57,66^. The internal standards used were d_4_-succinic acid (10 µg/extraction) for GC-MS analysis and genistein (1.25 µg/extraction) for LC-MS analysis. Aliquots of the polar and non-polar phase were immediately dried down after extraction. The total number of samples for the juvenile leaf set was 158 and 109 for the pre-flowering leaf set.

### Metabolite profiling with GC-MS

Dried aliquots for GC-MS analysis were derivatised as previously described with methoxymation and silylation derivatisation^67^, injected using a 10:1 split mode and analysed with a heat gradient from 70 to 325 °C^66^. Automated Mass Spectral Deconvolution and Identification System (AMDIS v2.71, NIST) was used to create an in-house library based on authentic standards and NIST’11 MS library (National Institute of Standards and Technology, USA). Peak convolution was performed with AMDIS in batch mode for each sample set and peak identification conducted according to metabolomics reporting guidelines^68,69^. Quantification of identified metabolites was expressed relative to the internal standard and sample weight (µg/g dry tissue).

### Untargeted analysis with LC-MS

Dried aliquots for LC-MS analysis were resuspended in methanol/water (1:1, v/v) and filtered using syringe filter (nylon, 0.45 µm). Separation of compounds was based on a previously published LC-MS method with modification of solvents A (water and 0.1% formic acid) and B (acetonitrile and 0.1% formic acid)^57^. Peak picking, alignment and identification was performed based on R package metaMS^70,71^ with a retention time window match set to 0.5 min. Peak identification was based on m/z difference 0.005 and retention difference 0.3 min to an in-house library with authentic standards. As described for GC-MS analysis, identified metabolites were relatively expressed.

### Targeted analysis with UPLC-DAD

For analysis of carotenoids and chlorophylls, the aliquot of the non-polar phase was resuspended in ethyl acetate/acetonitrile (1:9) and analysed as previously described^67^ with a composition gradient starting at 30% methanol/water (50:50, v/v) to 99% ethyl acetate/acetonitrile (25:75, v/v). The UV/Vis spectrum was continuously monitored from 250–600 nm. Metabolites were identified through retention time and UV/visible light spectrum compared to authentic standards, followed by a total quantification with dose-response curves^72^.

### Data processing and statistical analysis

All data was analysed using XLSTAT add-ins^73^ within Microsoft Excel, except PCA which was performed and visualised with Simca P 13.0.3.0 (Umetrics, Sweden). Metabolite data was rescaled from 0 to 1 (variable transformation) to remove biased of actual concentration levels (µg/g dry weight), which varied greatly between the different platforms. Differential expression (nonparametric) was performed with Benjamini-Yekutieli post-hoc correction on all replicates. Before the RV coefficient and Mantel test, the groups were averaged based on accession or genome group for each growth stage separately. The contribution of observation from PCAs was transformed with Spearman correlation into a correlation matrix. The *p*-value computation for the RV coefficient included 5000 permutations and 10000 permutations for the Mantel test with Spearman correlation. Hierarchical clustering was performed via Ward clustering in Simca P 13.0.3.0 (Umetrics, Sweden).

## Supplementary information

Dataset 1
