# Supplementary material for: Metabolite profiling characterises chemotypes of Musa diploids and triploids at juvenile and pre-flowering growth stages
Source: Sci Rep. 2019 Mar 15;9:4657. doi: 10.1038/s41598-019-41037-z (PMC6420674; doi:10.1038/s41598-019-41037-z)
Supplement: Supplementary file 1 — Dataset 1 [file 41598_2019_41037_MOESM1_ESM.pdf]

Supplementary Information

Metabolite profiling characterises chemotypes of *Musa* diploids and triploids at juvenile and pre-flowering growth stages

Margit Drapal[1], Elisabete Barros de Carvalho[1], Mathieu Rouard[2], Delphine Amah[3], Julie Sardos[2], Ines Van den Houwe[3], Allan Brown[4], Nicolas Roux[2], Rony Swennen[4,5,6] and Paul D.Fraser[1*]

[1] School of Biological Sciences, Royal Holloway, University of London, Egham Hill, Egham, Surrey, TW20 0EX, UK.

[2] Bioversity France, Parc Scientifique Agropolis II, 34397 Montpellier Cedex 5, France.

[3] International Institute of Tropical Agriculture, Ibadan, Nigeria

[4] International Institute of Tropical Agriculture, Arusha, Tanzania

[5] Bioversity International, W. De Croylaan 42, 3001 Heverlee, Belgium

[6] Department of Biosystem, KU Leuven University, Oude Markt 13 - bus 5005, 3000 Leuven, Belgium

Supplementary table S1. Library of metabolites detected in *Musa* species.

Supplementary Table S2. Metabolites detected in juvenile leaves.

Supplementary Table S3. Metabolites detected in pre-flowering leaves.

Supplementary Figure S4. Pathway displays showing ratio differences between wild and cultivated species with the same genetic background.

Supplementary Table S5. Differential expression of metabolites detected in juvenile leaves.

Supplementary Table S6. Differential expression of metabolites detected in pre-flowering leaves.

Supplementary Table S1. Library of metabolites detected in *Musa* by UPLC-DAD, GC-MS and LC-MS.

Metabolites	Analytical platform	Identification features (retention time (RT) in min.) RT (GC/MS)	RI (GC/MS)	RT (LC/MS)	m/z [M-H] (LC/MS)	RT (UPLC-DAD)	Identification Extract	Level[b]	Standard[c]	Compound class
**Alanine**	GC/MS	11.3	1107.9				Polar	1	Yes	Amino acid
**Disaccharide 47.6min**	GC/MS	48.4	2544.6				Polar	1	Yes	Sugar
**Erythronic acid/Threonic acid**	GC/MS	25.1	1571.3				Polar	1	Yes	Cell wall
**Fructose**	GC/MS	33.9	1874.2				Polar	1	Yes	Sugar
**Fumaric acid**	GC/MS	19.0	1346.8				Polar	1	Yes	TCA cycle
**GABA**	GC/MS	24.9	1547.5				Polar	1	Yes	Amino acid
**Glucose**	GC/MS	34.6	1910.4				Polar	1	Yes	Sugar
**Glucose/Galactose**	GC/MS	35.9	1882.2				Polar	1	Yes	Sugar
**Glutamine**	GC/MS	22.7	1471.3				Polar	1	Yes	Amino acid
**Glyceric acid**	GC/MS	18.2	1334.8				Polar	1	Yes	Cell wall
**Glycerol**	GC/MS	16.4	1278.2				Polar	1	Yes	Cell wall
**Glycerol galactose**	GC/MS	53.1	2755.3				Polar	1	Yes	Cell wall
**Glycerol-3-Phosphate**	GC/MS	31.0	1762.3				Polar	1	Yes	Cell wall
**Glycine**	GC/MS	17.3	1300.4				Polar	1	Yes	Amino acid
**Isoleucine**	GC/MS	17.0	1293.8				Polar	1	Yes	Amino acid
**Leucine**	GC/MS	17.4	1301.9				Polar	1	Yes	TCA cycle
**Mannose**	GC/MS	34.2	1887.7				Polar	1	Yes	Sugar
**Oxalic acid**	GC/MS	58.9	3125.2				Polar	1	Yes	Sugar
**Putrescine**	GC/MS	30.7	1752.3				Polar	1	Yes	Amino acid
**Pyroglutamic acid** [a]	GC/MS	24.1	1515.4				Polar	1	Yes	Amino acid
**Serine**	GC/MS	17.6	1259.7				Polar	1	Yes	Amino acid
**Valine**	GC/MS	13.9	1216.0				Polar	1	Yes	Amino acid
**260nm(II)** [d]	LC/MS			5.9	662.1		Polar	3	No	Phenylpropanoid
**260nm(III)** [d]	LC/MS			9.8	315.1		Polar	3	No	Phenylpropanoid
**260nm(IX)** [d]	LC/MS			13.9	443.2		Polar	3	No	Phenylpropanoid
**260nm(VII)** [d]	LC/MS			13.7	431.2		Polar	3	No	Phenylpropanoid
**260nm(VIII)** [d]	LC/MS			13.6	675.2		Polar	3	No	Phenylpropanoid
**260nm(XI)** [d]	LC/MS			14.3	755.2		Polar	3	No	Phenylpropanoid
**260nm(XII)** [d]	LC/MS			15.0	739.2		Polar	3	No	Phenylpropanoid
**260nm(XIV)** [d]	LC/MS			16.1	623.2		Polar	3	No	Phenylpropanoid
**260nm(XIX)** [d]	LC/MS			18.7	605.2		Polar	3	No	Phenylpropanoid
**260nm(XVII)** [d]	LC/MS			10.7	329.1		Polar	3	No	Phenylpropanoid
**4-Hydroxyproline**	LC/MS			1.4	130.0		Polar	1	Yes	Amino acid
**Abscisic acid**	LC/MS			18.5	263.1		Polar	1	Yes	Isoprenoid
**a-methyl-Serine**	LC/MS			1.4	118.1		Polar	1	Yes	Amino acid
**Arabinose/Ribose**	LC/MS			1.7	195.1		Polar	1	Yes	Sugar
**Arginine**	LC/MS			1.2	173.1		Polar	1	Yes	Amino acid
**Asparagine**	LC/MS			1.3	131.1		Polar	1	Yes	Amino acid
**Astragalin**	LC/MS			17.1	447.1		Polar	1	Yes	Phenylpropanoid
**AstragalinII**	LC/MS			17.4	447.1		Polar	1	Yes	Phenylpropanoid
**Caffeic acid**	LC/MS			12.8	179.0		Polar	1	Yes	Phenylpropanoid
**Caffeoyl-malate**	LC/MS			13.9	295.1		Polar	1	Yes	Phenylpropanoid
**Catechin**	LC/MS			10.6	289.1		Polar	1	Yes	Phenylpropanoid
**Chlorogenic acid**	LC/MS			12.7	353.1		Polar	1	Yes	Phenylpropanoid
**Cryptochlorogenic acid**	LC/MS			16.1	353.1		Polar	1	Yes	Phenylpropanoid
**Epiafzelechin**	LC/MS			14.9	273.1		Polar	1	Yes	Phenylpropanoid
**Epicatechin**	LC/MS			13.5	289.1		Polar	1	Yes	Phenylpropanoid
**Ferulic acid**	LC/MS			15.6	193.1		Polar	1	Yes	Phenylpropanoid
**Gallocatechin**	LC/MS			9.5	305.1		Polar	1	Yes	Phenylpropanoid
**Gentiobiose**	LC/MS			2.0	341.1		Polar	1	Yes	Sugar
**Glucose/Mannose/Galactose/Sorbose**	LC/MS			1.7	179.1		Polar	1	Yes	Sugar
**Glucuronic acid**	LC/MS			1.8	193.0		Polar	1	Yes	Sugar
**Glutamic acid**	LC/MS			1.7	146.0		Polar	1	Yes	Amino acid
**Guanine**	LC/MS			1.6	150.1		Polar	1	Yes	Nucleotide
**Histidine**	LC/MS			1.6	154.1		Polar	1	Yes	Amino acid
**Homoserine**	LC/MS			1.4	118.1		Polar	1	Yes	Amino acid
**Isocitric acid**	LC/MS			3.0	191.0		Polar	1	Yes	TCA cycle
**Isoferulic acid**	LC/MS			15.0	193.1		Polar	1	Yes	Phenylpropanoid
**Kaempferol-3-O-rutinoside**	LC/MS			15.7	593.2		Polar	1	Yes	Phenylpropanoid
**Kaempferol-rutinoside**	LC/MS			16.6	593.2		Polar	1	Yes	Phenylpropanoid
**Lysine**	LC/MS			1.4	145.1		Polar	1	Yes	Amino acid
**m-Coumaric acid**	LC/MS			15.2	163.0		Polar	1	Yes	Phenylpropanoid
**Myricitrin**	LC/MS			14.8	463.1		Polar	1	Yes	Phenylpropanoid
**Neochlorogenic acid**	LC/MS			10.8	353.1		Polar	1	Yes	Phenylpropanoid
**o-Coumaric acid**	LC/MS			19.1	163.1		Polar	1	Yes	Phenylpropanoid
**p-Coumaric acid**	LC/MS			13.4	163.0		Polar	1	Yes	Phenylpropanoid
**Quercetin**	LC/MS			20.9	301.0		Polar	1	Yes	Phenylpropanoid
**Quercetin-glucoside**	LC/MS			16.0	463.1		Polar	1	Yes	Phenylpropanoid
**Quinic acid**	LC/MS			1.7	191.1		Polar	1	Yes	Phenylpropanoid
**Rhamnose**	LC/MS			1.7	209.1		Polar	1	Yes	Sugar
**Ribitol/Xylitol**	LC/MS			1.6	151.1		Polar	1	Yes	Sugar
**Rutin**	LC/MS			15.0	609.1		Polar	1	Yes	Phenylpropanoid
**RutinII**	LC/MS			15.6	609.1		Polar	1	Yes	Phenylpropanoid
**Sedoheptulose**	LC/MS			1.8	237.1		Polar	1	Yes	Sugar
**Shikimic acid**	LC/MS			2.0	173.1		Polar	1	Yes	Organic acid
**Sinapic acid**	LC/MS			16.3	223.1		Polar	1	Yes	Phenylpropanoid
**Trehalose**	LC/MS			1.6	387.1		Polar	1	Yes	Sugar
**Tryptophan**	LC/MS			9.5	203.1		Polar	1	Yes	Amino acid
**Tyrosine**	LC/MS			3.1	180.1		Polar	1	Yes	Amino acid
**Aspartic acid**	LC/MS, GC/MS	23.1	1522.0	1.4	132.0		Polar	1	Yes	Amino acid
**Citric acid**	LC/MS, GC/MS	32.5	1821.5	3.4	191.0		Polar	1	Yes	TCA cycle
**Dopamine**	LC/MS, GC/MS	39.6	2103.6	1.6	152.1		Polar	1	Yes	Dopamine
**Malic acid**	LC/MS, GC/MS	23.3	1491.5	2.0	133.0		Polar	1	Yes	TCA cycle
**myo-Inositol**	LC/MS, GC/MS	39.6	2110.9	1.6	225.1		Polar	1	Yes	Sugar
**Phenylalanine**	LC/MS, GC/MS	25.1	1541.5	7.0	164.1		Polar	1	Yes	Amino acid
**Proline**	LC/MS, GC/MS	17.5	1311.4	1.7	114.0		Polar	1	Yes	Amino acid
**Sucrose**	LC/MS, GC/MS	51.1	2676.9	2.0	387.1		Polar	1	Yes	Sugar
**Threonine**	LC/MS, GC/MS	18.9	1386.1	1.4	118.1		Polar	1	Yes	Amino acid
**Turanose**	LC/MS, GC/MS	50.1	2611.2	1.6	387.1		Polar	1	Yes	Sugar
**Antheraxanthin**	UPLC-PDA					1.0	Non-polar	1	Yes	Isoprenoid
**b-Carotene**	UPLC-PDA					5.4	Non-polar	1	Yes	Isoprenoid
**b-Cryptoxanthin-5,6-epoxide**	UPLC-PDA					2.0	Non-polar	1	Yes	Isoprenoid
**Chlorophyll a**	UPLC-PDA					4.3	Non-polar	1	Yes	Isoprenoid
**Chlorophyll a' (C-10 epimer)**	UPLC-PDA					4.4	Non-polar	1	Yes	Isoprenoid
**Chlorophyll b**	UPLC-PDA					3.7	Non-polar	1	Yes	Isoprenoid
**Chlorophyll b' (C-10 epimer)**	UPLC-PDA					3.9	Non-polar	1	Yes	Isoprenoid
**Lutein**	UPLC-PDA					1.4	Non-polar	1	Yes	Isoprenoid
**Neoxanthin**	UPLC-PDA					0.8	Non-polar	1	Yes	Isoprenoid
**Pheophytin a** [e]	UPLC-PDA					5.3	Non-polar	2	No	Isoprenoid
**Phylloquinone**	UPLC-PDA					4.5	Non-polar	1	Yes	Quinone
**Phytoene**	UPLC-PDA					5.9	Non-polar	1	Yes	Isoprenoid
**Quinone**	UPLC-PDA					2.3	Non-polar	2	No	Quinone
**Violaxanthin 13-cis**	UPLC-PDA					2.2	Non-polar	1	Yes	Isoprenoid
**Violaxanthin 9-cis**	UPLC-PDA					2.1	Non-polar	1	Yes	Isoprenoid
**Violaxanthin isomer1**	UPLC-PDA					0.7	Non-polar	1	Yes	Isoprenoid
**Violaxanthin isomer2**	UPLC-PDA					0.8	Non-polar	1	Yes	Isoprenoid

[a] *Derivatisation of glutamic acid produces the corresponding TMS derivatives in addition to TMS-pyroglutamic acid derivatives (Kanani H, Klapa MI (2007). Metabolic Engineering, 9: 39-51).*

[b] *Level of metabolite identification as in (Bino et al., 2004, Sumner et al., 2007) : (1) Identified compounds; (2) Putatively annotated compounds; (3) Putatively characterized compound classes; (4) Unknown compounds*

[c] *Identification confirmed by spectral and chromatographic properties comparison to authentic commercial standards*

[d] *Compound class identified by their UV/VIS spectra, specific to Musa leaf tissue.*

[e] *Identified by UV/Vis spectrum and the folowing publication: Edelenbos et al. (2001) J. Agric. Food Chem.,49:4768-4774.*

Supplementary Table S2. Metabolites detected in juvenile leaves. Values are calculated as µg/g dry weight relative to the internal standard.

Variety	Genotype	4-Hydroxyproline	Abscisic acid	Alanine	a-methyl-Serine/Homoserine/Threonine	Arabinose/Ribose	Arginine	Asparagine	Aspartic acid	Aspartic acid_GCMS	Caffeic acid	Caffeoyl-malate	Catechin
ITC0084	AAA	272.2	489.2	267.1	2783.0	5042.2	11704.3	8142.8	4839.7	0.000	679.1	1326.7	114.4
ITC0084	AAA	82.6	174.5	375.3	840.9	3675.6	7494.5	3807.3	2104.4	0.000	366.4	321.0	38.7
ITC0084	AAA	114.5	161.9	316.0	2234.5	2333.2	7043.5	6894.1	3527.0	325.1	530.5	378.8	76.9
ITC0084	AAA	89.2	135.4	120.2	1210.8	4714.1	8256.8	4231.4	2586.1	198.8	510.4	387.7	58.9
ITC0084	AAA	152.1	297.1	120.7	2001.1	4722.2	13860.2	6929.0	4717.0	0.000	874.1	623.4	77.7
ITC0084	AAA	102.8	180.6	106.8	1542.3	2567.5	6351.8	3866.9	3265.9	162.8	705.7	454.8	74.9
ITC0111	AAB	410.7	183.2	1120.2	1751.5	3157.1	3588.1	5487.7	1783.8	463.1	970.9	60.2	280.7
ITC0111	AAB	250.5	106.8	1050.6	855.5	1667.7	1338.9	2537.5	852.0	491.9	579.4	19.0	120.1
ITC0111	AAB	318.9	113.1	1190.6	1050.9	1924.5	1593.9	3267.9	1045.5	492.4	627.4	14.7	113.7
ITC0111	AAB	189.6	77.2	1175.0	616.8	1387.1	1014.0	2109.3	572.0	555.2	517.4	12.7	92.4
ITC0111	AAB	193.1	68.7	755.5	789.5	1522.3	833.8	2834.5	770.8	392.2	476.3	20.0	105.4
ITC0112	AAB	81.8	214.8	1405.4	1160.9	2579.8	1140.6	4389.3	2194.9	335.9	496.1	60.7	117.2
ITC0112	AAB	89.4	194.8	1516.0	1265.1	1976.9	2041.4	3737.6	1812.1	415.5	606.9	39.1	124.6
ITC0112	AAB	65.4	140.5	1334.6	769.6	2254.5	1149.8	2403.7	1589.3	364.8	547.8	40.7	63.9
ITC0112	AAB	59.4	191.1	717.4	1208.6	1984.3	1603.3	3979.6	1678.8	194.7	432.8	36.0	96.3
ITC0112	AAB	110.0	306.6	2818.0	1618.6	2810.2	3063.1	5980.3	2495.8	235.8	531.4	37.8	137.6
ITC0112	AAB	94.8	178.1	532.2	1177.5	2621.2	1563.0	4920.3	2053.8	140.1	575.8	68.7	102.2

Supplementary Table S3. Metabolites detected in pre-flowering leaves. Values are calculated as µg/g dry weight relative to the internal standard.

Variety	Genome	260nm(II)	260nm(III)	260nm(IX)	260nm(VII)	260nm(VIII)	260nm(XI)	260nm(XII)	260nm(XIII)	260nm(XIV)	260nm(XIX)	260nm(XVII)	Abscisic acid	Antheraxanthin	Arabinose/Ribose	Aspartic acid	Astragalin	AstragalinII	b-Carotene	b-Cryptoxanthin-5,6-epoxide
ITC0084	AAA	19.18	158.10	21.72	213.70	46.25	213.13	143.20	9.226	199.90	19.62	135.66	1.948	104.60	135.69	23.15	203.07	26.99	259.90	91.52
ITC0084	AAA	21.55	1026.17	47.70	472.17	92.73	233.49	142.00	14.30	227.33	15.36	181.50	5.855	111.25	134.13	12.93	143.15	25.95	262.93	91.88
ITC0084	AAA	35.69	122.15	23.67	270.92	68.03	224.58	165.74	7.645	238.07	22.40	181.35	5.078	113.69	85.95	16.77	148.78	37.09	270.83	92.18
ITC0084	AAA	25.66	103.96	21.65	169.76	39.74	210.11	135.09	9.670	99.54	19.56	102.58	4.214	107.21	90.90	17.24	252.10	33.59	193.80	93.98
ITC0084	AAA	23.28	55.09	19.31	149.99	42.44	154.13	109.29	3.003	126.63	15.98	93.65	4.371	101.81	62.71	12.94	229.30	22.50	364.38	86.32
ITC0084	AAA	16.54	941.12	42.29	395.79	86.68	234.81	152.11	10.93	199.75	21.30	149.80	14.20	106.82	176.82	18.12	292.41	27.90	453.92	91.18
ITC0111	AAB	23.60	362.88	22.38	326.33	49.32	128.93	193.96	8.854	155.40	75.11	119.67	4.633	104.80	51.16	20.41	295.85	36.61	275.69	94.72
ITC0111	AAB	22.90	506.89	52.67	562.01	68.23	137.55	204.46	7.069	277.62	57.57	147.36	4.508	103.18	92.62	33.65	565.36	47.72	290.53	93.07
ITC0111	AAB	27.53	515.37	34.24	486.92	65.83	174.89	255.45	14.08	213.44	67.57	146.46	4.898	103.27	45.92	20.87	468.95	53.85	294.20	92.68
ITC0112	AAB	26.81	443.16	38.23	399.16	83.94	180.11	293.29	13.09	216.28	50.56	160.99	4.473	108.03	85.74	28.64	786.00	65.61	324.63	94.25
ITC0112	AAB	34.77	449.70	24.09	300.40	54.99	214.82	385.13	7.194	191.91	70.93	210.95	4.819	107.15	54.52	34.38	658.29	53.34	282.69	94.43
ITC0112	AAB	33.18	448.40	35.41	379.42	78.15	207.88	366.77	11.91	211.41	66.86	200.96	4.750	108.71	79.50	33.23	760.47	63.16	284.76	92.96
ITC0121	AAB	37.28	754.99	26.45	389.13	70.49	132.83	143.37	11.15	247.78	60.82	137.20	7.048	102.61	78.61	59.72	305.14	74.81	265.36	93.44
ITC0121	AAB	25.77	819.40	15.77	260.09	43.62	83.79	75.28	5.724	76.11	22.09	53.88	1.850	100.42	51.27	32.63	187.51	51.74	197.21	89.93
ITC0121	AAB	27.63	231.47	15.54	270.10	18.79	95.65	78.84	3.179	103.32	17.82	36.59	0.922	104.57	41.52	17.68	183.70	21.33	252.71	90.06
ITC0121	AAB	26.24	1154.73	35.49	465.55	70.52	93.77	89.60	8.097	304.06	36.74	102.72	4.532	103.17	110.60	40.45	282.47	83.31	271.89	92.91
ITC0121	AAB	27.67	778.57	21.79	408.73	71.34	135.00	135.41	7.759	273.10	57.47	126.80	6.852	103.63	88.73	64.97	373.66	71.04	285.65	92.16
ITC0123	ABB	24.49	427.21	28.18	219.26	58.75	110.72	182.53	38.19	28.18	21.69	201.60	3.459	99.41	31.50	24.15	85.05	20.09	120.47	85.00
ITC0123	ABB	22.03	392.75	27.83	316.52	64.89	217.50	236.35	56.58	100.94	26.36	249.17	4.036	105.42	40.93	26.91	135.61	30.04	113.60	90.25
ITC0123	ABB	24.00	420.32	28.11	297.07	63.67	196.15	225.59	52.91	86.40	25.42	239.66	3.920	104.22	39.05	26.36	125.50	28.05	119.10	89.20
ITC0245	AB	20.13	176.98	5.365	433.56	39.74	129.01	70.69	8.580	115.97	20.59	49.02	2.950	104.75	37.29	6.350	76.64	38.82	284.73	90.88
ITC0245	AB	22.01	225.03	3.383	340.07	31.09	141.00	94.81	10.78	75.06	25.65	48.96	32.70	106.69	67.12	11.07	146.00	77.16	428.07	89.85
ITC0245	AB	20.21	214.08	1.303	237.28	19.23	136.96	62.95	5.761	79.43	2.697	33.40	1.372	105.06	46.46	5.547	36.44	27.25	603.54	85.44
ITC0249	AAw	26.24	189.59	49.47	93.93	25.77	110.75	78.30	6.417	1401.51	36.32	167.71	6.782	104.13	73.19	23.29	843.73	81.78	290.01	89.57
ITC0249	AAw	29.13	2221.89	51.85	111.14	41.41	151.46	90.25	7.397	1473.22	32.29	253.97	5.975	103.92	105.12	17.06	523.10	96.02	218.54	86.91
ITC0249	AAw	37.48	1760.00	63.97	101.29	37.29	161.74	100.16	8.208	1602.36	41.90	244.70	12.41	103.87	83.67	27.89	493.11	103.11	211.20	87.25
ITC0250	AAw	16.92	776.12	36.10	76.79	42.04	308.83	164.84	3.400	1422.82	32.71	219.53	7.213	118.18	60.02	27.29	396.33	20.04	482.11	97.86
ITC0250	AAw	19.67	745.93	35.10	69.58	21.57	247.33	125.16	11.38	92.28	31.52	129.76	2.945	105.38	60.12	19.36	248.24	25.17	289.33	93.18
ITC0250	AAw	18.59	801.44	35.88	76.54	21.29	257.69	127.28	11.69	94.54	33.07	170.38	3.221	106.38	52.14	23.71	292.01	16.23	294.34	93.55
ITC0250	AAw	25.04	2930.73	41.49	157.87	60.50	337.47	189.48	1.516	1772.50	29.77	318.56	11.50	105.14	96.10	29.17	212.25	11.48	196.61	89.45
ITC0250	AAw	26.42	1171.36	49.92	102.14	29.08	361.81	176.01	16.31	141.76	43.92	240.94	5.065	106.37	79.63	38.07	398.43	25.55	277.43	93.97
ITC0253	AAw	16.03	279.61	32.66	27.59	11.75	211.56	38.99	8.967	78.20	131.99	380.97	2.684	100.62	70.17	40.90	375.61	37.15	261.73	90.51
ITC0253	AAw	19.66	389.17	74.03	55.38	16.24	226.64	47.86	10.79	115.90	135.74	381.92	4.199	104.53	73.59	62.04	395.49	56.98	262.82	93.23
ITC0253	AAw	21.68	98.16	39.62	25.88	10.95	207.05	48.98	8.365	81.56	106.93	378.62	3.355	104.61	54.43	77.28	366.48	38.87	279.94	91.29
ITC0277	AAA	31.28	1013.84	24.42	198.53	20.67	288.67	156.37	15.28	437.51	74.55	214.52	2.161	102.60	147.54	38.07	151.25	41.55	292.19	93.94
ITC0277	AAA	21.43	568.62	19.56	151.04	15.71	253.87	161.12	5.250	252.39	78.61	132.56	1.452	102.49	68.10	23.45	192.91	42.33	118.59	87.51
ITC0277	AAA	30.85	664.64	28.42	115.82	12.97	213.58	179.52	9.778	220.52	54.18	138.62	15.91	101.68	46.41	9.015	186.73	23.00	251.53	92.01
ITC0312	AAc	47.19	76.32	47.90	76.48	18.23	351.02	173.03	8.218	420.27	18.40	130.48	8.419	102.54	85.47	16.41	162.31	23.60	119.67	90.08
ITC0312	AAc	35.06	76.07	58.37	60.26	10.19	306.94	133.55	5.983	349.87	15.39	144.04	8.202	99.57	47.29	9.933	109.81	17.44	369.20	90.48
ITC0312	AAc	30.62	59.65	74.77	65.71	6.439	281.97	92.76	5.640	353.56	18.55	201.21	15.80	101.95	42.65	10.71	87.69	15.32	319.31	90.40
ITC0361	ABB	36.81	431.95	40.85	290.13	56.12	169.58	170.40	20.87	96.27	60.29	196.67	3.446	107.53	111.61	32.18	108.85	47.08	401.83	98.07
ITC0361	ABB	20.08	138.15	20.63	165.53	47.10	133.70	136.76	21.62	59.38	24.28	114.10	1.837	108.60	113.95	32.77	112.01	37.85	613.67	103.96
ITC0361	ABB	40.99	505.37	45.90	321.26	58.37	178.55	178.81	17.90	105.48	69.28	217.30	3.849	100.13	102.24	29.81	96.20	49.39	274.87	91.23
ITC0472	ABB	3.700	6.917	0.925	11.16	0.322	18.66	36.72	1.870	22.36	0.845	6.193	5.288	106.71	8.345	11.14	17.55	9.068	273.27	93.84
ITC0472	ABB	22.78	113.41	3.522	85.38	20.20	70.29	292.66	4.653	26.86	62.63	247.70	5.801	115.79	19.20	17.76	17.49	4.254	307.59	94.30
ITC0472	ABB	22.39	77.35	3.474	85.81	24.78	106.50	274.13	5.159	25.04	48.16	301.35	4.961	113.46	22.00	19.64	47.98	3.866	292.56	95.35
ITC0587	AAB	23.91	586.22	35.26	250.20	40.58	311.18	104.49	11.25	130.02	30.56	188.81	4.958	106.63	64.36	11.91	14.76	18.95	267.57	93.62
ITC0587	AAB	22.85	614.28	31.53	207.73	35.79	329.83	175.24	10.97	165.40	79.20	368.12	3.808	104.69	113.04	23.91	33.53	25.75	275.74	93.53
ITC0587	AAB	44.42	1204.67	52.62	377.29	61.98	588.08	285.94	18.29	302.07	76.04	854.80	8.324	106.54	107.03	41.89	46.73	56.29	265.36	93.13
ITC0649	AAB	25.92	1197.60	19.14	313.47	57.02	197.96	241.88	40.05	193.57	64.63	427.73	7.115	103.81	41.61	12.17	191.60	40.75	272.03	92.79
ITC0649	AAB	20.80	716.28	10.05	179.84	40.58	159.22	183.12	23.70	155.89	62.74	313.91	4.757	101.96	30.53	17.44	215.89	45.38	273.64	91.45
ITC0649	AAB	14.47	586.06	9.755	183.73	38.79	126.28	129.73	18.14	165.45	47.19	199.47	3.486	102.17	53.33	14.31	225.64	35.38	262.53	93.27
ITC0653	AAc	21.64	369.00	36.52	531.70	15.77	483.53	254.07	24.76	320.50	55.23	303.72	3.538	101.17	48.16	17.17	237.74	57.70	263.93	91.37
ITC0653	AAc	16.08	367.77	44.50	418.92	8.819	422.80	196.10	18.03	266.81	46.20	335.29	3.447	102.31	26.65	10.39	160.84	42.64	267.56	93.03
ITC0653	AAc	18.90	289.36	46.78	579.76	9.959	444.20	176.48	23.35	323.88	66.56	424.27	6.815	105.71	43.44	18.51	189.84	50.71	280.91	92.78
ITC0654	AAA	20.75	1046.47	26.84	116.66	17.13	276.60	182.83	10.87	179.01	66.30	461.56	4.222	107.39	49.19	27.93	249.91	24.41	279.20	93.20
ITC0654	AAA	33.76	513.39	41.68	203.53	17.19	417.08	287.22	14.58	218.55	95.23	705.11	7.435	107.25	59.62	37.19	312.37	34.74	274.77	94.34
ITC0654	AAA	20.84	858.34	17.17	125.01	12.51	251.49	177.24	11.40	136.96	63.36	515.57	4.999	110.34	39.66	26.85	169.86	13.22	285.26	93.67
ITC0659	ABB	22.63	723.78	2.180	263.56	43.77	255.86	219.47	5.362	30.01	66.05	177.45	4.784	102.10	35.60	14.39	279.61	17.49	268.78	92.34
ITC0659	ABB	23.47	787.75	2.288	271.34	53.02	250.10	239.48	6.371	33.71	69.56	190.65	5.640	105.87	56.42	18.15	265.32	22.64	285.23	92.07
ITC0659	ABB	20.75	790.72	3.205	250.02	58.50	238.30	249.20	7.977	39.35	75.10	209.83	7.803	104.16	54.09	17.85	260.96	27.64	278.07	91.89
ITC0662	AAA	11.28	724.64	15.75	97.56	16.16	80.54	44.78	5.770	43.19	39.72	198.38	5.400	104.15	25.63	12.57	10.48	2.310	291.38	93.50
ITC0662	AAA	19.94	338.87	42.43	111.98	17.29	207.83	82.64	8.006	115.65	50.88	244.24	4.882	101.24	76.13	29.03	127.96	33.46	287.07	92.46
ITC0662	AAA	10.80	438.83	64.96	107.81	16.48	139.15	48.10	10.83	107.71	36.30	194.45	3.582	101.92	166.10	76.18	173.86	18.02	277.03	94.77
ITC0767	ABB	20.99	1024.43	37.98	274.59	45.64	115.68	143.87	70.63	106.92	11.37	64.67	9.001	108.02	59.38	15.74	50.54	23.74	562.42	98.64
ITC0767	ABB	30.24	915.16	51.46	327.73	54.87	126.36	155.23	64.06	121.19	9.550	91.15	4.653	104.97	86.34	17.09	46.80	26.07	261.22	94.86
ITC0767	ABB	37.02	1829.72	50.62	414.20	71.82	162.25	217.10	45.85	91.28	11.73	116.90	2.057	109.36	74.58	21.33	40.02	18.08	283.45	92.55
ITC0767	ABB	33.82	1668.73	48.10	386.29	66.58	152.94	202.46	65.68	103.80	11.66	106.46	7.612	108.45	71.54	20.21	48.44	22.60	244.01	97.19
ITC0767	ABB	23.28	920.95	61.03	392.01	71.18	120.27	158.90	59.09	162.29	9.130	97.71	4.199	106.62	118.26	20.60	67.13	24.55	266.45	95.11
ITC0767	ABB	25.71	908.76	48.68	310.04	51.74	114.61	135.63	53.56	124.88	7.790	83.73	5.258	106.02	62.28	18.51	57.84	24.19	274.48	94.82
ITC0769	AAB	16.95	654.37	5.543	124.89	30.36	143.07	200.06	7.908	95.34	54.20	133.83	5.056	103.69	50.86	15.02	246.18	20.21	281.15	92.25
ITC0769	AAB	22.19	653.64	7.880	182.93	41.69	234.03	288.42	17.58	143.80	53.16	84.67	5.252	105.65	79.68	34.21	304.90	38.77	270.15	92.49
ITC0769	AAB	15.11	531.55	7.500	226.37	40.38	178.57	205.62	14.56	180.56	45.13	74.77	3.565	106.13	48.43	36.03	261.65	44.24	279.02	91.63
ITC1120	BB	36.84	218.34	6.591	372.42	65.40	300.10	227.44	71.10	99.76	22.48	228.10	4.850	100.52	41.61	17.55	132.15	65.82	214.10	85.95
ITC1120	BB	38.06	142.38	7.705	577.83	55.28	281.10	272.97	86.28	140.27	12.41	259.20	7.975	103.57	68.56	29.87	153.57	83.24	253.30	89.49
ITC1120	BB	30.21	80.90	11.81	397.44	76.89	265.78	199.79	62.36	121.83	7.819	166.52	9.129	105.26	73.57	28.98	111.63	64.19	211.03	91.14
ITC1121	AA	18.00	365.08	15.05	38.93	9.308	127.95	77.37	2.509	490.98	17.51	125.72	1.489	100.07	20.75	24.97	168.08	8.221	289.09	90.75
ITC1121	AA	14.72	700.49	12.79	43.69	7.581	124.79	86.08	2.763	539.34	26.05	124.26	1.438	108.91	22.32	17.84	236.21	11.91	250.54	100.27
ITC1121	AA	15.54	806.05	30.80	86.52	14.35	218.92	104.66	10.90	885.56	37.93	145.14	3.199	107.77	26.67	18.29	254.58	11.54	164.88	94.48
ITC1122	AAA	27.25	3512.20	34.52	276.49	74.23	484.00	216.65	4.522	1037.63	62.35	387.23	4.441	109.54	58.83	14.13	397.05	35.56	146.11	101.39
ITC1122	AAA	20.71	1147.26	22.76	96.60	46.69	259.83	184.61	4.724	541.53	62.78	218.95	2.671	109.28	33.54	13.85	466.63	38.41	149.30	90.90
ITC1122	AAA	28.88	4103.15	37.46	321.44	81.12	540.01	224.66	3.712	1161.59	60.65	429.28	4.883	107.84	65.15	14.20	118.57	24.15	206.56	94.09
ITC1177	AAw	14.05	368.21	4.124	37.40	11.31	189.53	95.69	12.37	109.56	22.73	170.48	3.563	108.64	25.38	12.99	31.02	6.468	291.12	93.39
ITC1177	AAw	17.33	459.38	5.953	45.29	13.79	213.53	119.58	15.53	125.38	26.53	199.31	3.993	110.91	27.28	14.07	32.06	8.230	277.82	93.68
ITC1177	AAw	16.84	409.65	8.982	49.48	16.14	213.41	127.94	13.57	192.19	26.24	296.87	10.63	108.07	24.70	16.51	41.19	10.93	393.47	93.79
ITC1177	AAw	17.01	1261.57	13.79	84.43	26.28	251.49	118.73	12.37	194.50	30.25	454.35	3.845	109.77	63.43	17.13	42.77	8.945	309.84	94.03
ITC1177	AAw	21.11	796.28	13.67	80.28	19.58	194.45	67.68	6.083	216.98	15.49	283.90	2.210	104.86	31.89	15.45	40.70	8.380	171.42	93.87
ITC1177	AAw	15.00	821.87	11.77	63.15	31.83	216.13	88.35	8.895	200.80	21.47	353.78	3.641	108.64	69.62	18.92	27.53	6.929	497.11	92.62
ITC1177	AAw	19.09	561.62	7.725	53.79	14.45	235.36	135.14	15.71	153.91	30.83	293.78	4.685	107.43	30.09	20.13	48.32	10.35	283.08	93.20
ITC1187	AAc	34.31	1977.41	85.10	179.92	31.93	125.09	164.55	13.49	108.12	50.22	166.90	6.231	103.90	54.03	21.81	883.15	62.40	206.27	87.44
ITC1187	AAc	41.12	3991.58	135.52	328.90	52.53	152.38	192.63	15.79	181.77	72.10	340.40	12.54	103.85	52.87	21.41	1360.91	77.00	202.73	86.89
ITC1187	AAc	38.68	3269.34	117.44	275.48	45.14	142.60	182.56	14.96	155.36	64.26	278.19	10.28	103.78	53.62	21.66	1189.60	71.76	431.10	88.68
ITC1325	AAB	29.78	1114.13	24.45	388.18	34.07	107.67	122.28	12.46	273.77	44.80	152.18	7.990	109.76	83.27	45.04	384.51	70.26	492.82	104.07
ITC1325	AAB	25.30	1040.34	38.91	595.48	67.69	73.83	97.85	8.690	221.52	51.24	151.66	5.834	102.23	42.30	19.84	427.84	60.82	282.25	92.78
ITC1325	AAB	29.12	1629.32	59.28	673.28	58.36	120.63	155.97	11.01	259.57	75.16	199.88	7.084	103.15	66.47	29.51	499.40	71.71	278.98	92.42
ITC1441	AAB	14.10	240.64	1.242	127.41	30.81	143.78	109.79	10.90	61.74	29.60	106.94	1.381	101.93	35.64	11.35	61.74	18.70	126.78	84.67
ITC1441	AAB	21.66	277.02	3.858	328.01	66.13	229.37	194.65	32.71	244.45	54.69	128.10	5.036	109.40	40.99	30.04	177.51	34.79	102.35	92.97
ITC1441	AAB	19.30	284.57	2.771	313.18	53.90	190.37	155.34	15.43	127.84	61.20	97.39	4.410	104.72	23.45	11.75	120.49	24.15	257.91	92.42
ITC1441	AAB	23.32	453.14	3.767	322.92	51.81	247.37	211.34	39.34	162.11	59.69	142.40	3.511	108.90	41.41	13.22	184.36	30.63	219.37	100.16
ITC1441	AAB	21.62	519.27	4.445	309.04	60.60	229.28	232.69	18.48	113.21	73.60	114.50	4.995	104.61	47.44	13.32	126.64	23.64	284.47	92.00
ITC1441	AAB	10.80	301.72	0.898	163.20	40.22	132.20	132.86	13.83	76.44	34.19	131.42	1.177	100.61	38.03	11.41	76.75	24.10	122.03	88.76
ITC1441	AAB	20.51	450.69	2.041	249.80	51.88	185.98	164.71	18.56	164.01	64.81	154.70	4.366	104.41	38.28	14.35	105.56	28.03	282.05	91.62
ITC1483	ABB	30.76	143.76	33.40	214.46	28.89	201.19	184.36	42.32	66.98	15.61	135.65	2.942	106.56	38.39	34.32	117.86	26.60	451.15	99.00
ITC1483	ABB	24.15	380.14	30.85	183.33	30.83	183.60	168.84	38.38	44.56	19.60	154.64	3.182	105.37	39.91	29.78	86.50	25.02	318.14	95.00
ITC1483	ABB	28.65	234.95	37.68	206.28	37.36	200.20	157.46	26.92	55.39	15.03	117.04	4.657	106.92	36.05	24.15	114.69	21.60	279.67	92.78
ITC1483	ABB	23.59	232.68	43.68	246.19	42.61	220.43	189.25	23.70	75.62	15.60	131.87	5.023	109.74	77.36	47.30	155.62	22.37	277.50	95.03
ITC1483	ABB	29.01	159.52	44.04	235.09	33.40	254.74	222.48	37.07	66.78	16.27	134.26	4.883	105.12	50.26	35.80	142.81	33.50	297.74	87.98
ITC1483	ABB	14.11	305.84	25.18	220.92	35.55	140.33	139.67	37.57	73.57	30.87	96.59	1.243	107.27	42.17	20.26	64.43	24.12	503.98	90.89
ITC1483	ABB	41.67	949.04	57.68	417.20	68.99	382.41	272.82	42.77	129.81	24.30	284.91	8.217	108.70	67.58	48.65	181.35	38.19	296.29	92.11

Variety	Caffeic acid	Catechin	Chlorogenic acid	Chlorophyll a	Chlorophyll a'	Chlorophyll b	Chlorophyll b'	Citric acid	Cryptochlorogenic acid	Epiafzelechin	Epicatechin	Gallocatechin	Gentiobiose	Glucose/Mannose/Galactose/Sorbose	Glucuronic acid	Glutamic acid
ITC0084	31.75	4.663	41.27	3573.07	346.91	1250.75	151.62	3797.48	2201.63	11.66	638.89	5.383	42.95	93.01	22.81	34.89
ITC0084	34.92	9.914	114.33	3529.20	464.82	1385.33	219.73	6940.88	2350.19	18.05	963.18	5.959	63.52	172.92	28.60	40.15
ITC0084	43.36	10.62	146.10	4596.43	639.62	1852.21	338.78	7873.66	2810.39	17.93	1140.86	7.087	57.91	92.14	26.71	53.82
ITC0084	44.35	6.731	160.48	6335.17	244.67	2064.00	147.90	12806.71	5477.19	13.52	783.06	3.539	47.64	78.14	24.30	31.02
ITC0084	34.50	8.100	142.10	8067.73	387.12	2586.51	287.71	13983.16	5481.62	14.10	916.59	4.313	42.18	46.04	21.58	32.61
ITC0084	38.10	9.634	142.16	8422.98	289.64	2458.61	198.03	14460.36	7055.28	22.21	1194.96	5.428	64.17	129.84	34.95	42.69
ITC0111	36.15	9.989	56.21	5677.06	368.26	1779.97	110.92	2871.51	1635.47	275.32	517.27	5.712	67.84	33.87	20.77	28.76
ITC0111	27.85	7.218	74.01	6028.13	371.96	1929.07	139.88	2088.63	1862.18	304.41	495.89	4.389	78.64	49.38	10.49	64.26
ITC0111	45.96	9.827	97.00	6580.30	354.12	1949.56	138.65	2949.98	1850.33	331.79	558.21	5.418	68.08	31.24	21.98	30.85
ITC0112	44.06	15.07	63.05	8742.11	526.23	2899.29	202.11	1688.59	3257.91	559.42	1044.97	5.406	83.51	33.53	9.910	87.04
ITC0112	66.73	17.15	111.38	6937.59	537.05	2283.72	155.49	3170.51	3220.53	516.59	1020.96	6.237	102.78	37.84	13.18	76.46
ITC0112	62.20	16.73	101.72	6862.05	404.61	2593.62	196.51	2874.24	3250.44	550.86	1040.17	6.071	98.93	36.98	12.52	84.93
ITC0121	43.15	13.30	141.32	4364.41	362.21	1417.51	126.42	2196.18	2093.99	401.11	688.07	8.043	90.78	123.58	12.98	115.42
ITC0121	31.02	7.852	104.51	2387.64	214.99	888.52	116.38	1896.26	4324.72	212.89	436.24	3.783	73.80	43.82	8.906	37.07
ITC0121	19.11	2.741	58.44	4936.66	168.90	1285.36	106.33	723.74	2968.25	72.31	78.98	1.981	40.25	48.79	3.533	39.14
ITC0121	32.65	9.687	59.86	4583.92	349.47	1492.73	103.89	2351.46	2510.37	334.25	647.45	4.962	80.94	60.54	8.615	66.14
ITC0121	24.78	13.22	69.77	5890.53	546.41	1945.65	203.73	2253.78	2344.92	461.25	763.84	6.226	73.05	100.86	9.078	138.82
ITC0123	30.85	20.47	102.82	3792.13	182.83	1263.89	61.68	2087.24	2781.89	4219.75	868.70	6.686	82.59	53.90	15.90	30.26
ITC0123	37.23	16.70	130.44	4802.42	210.69	1585.59	67.29	1658.40	2595.36	4726.70	743.69	5.887	88.11	52.70	14.31	32.90
ITC0123	35.95	19.72	124.92	4600.44	205.12	1521.27	66.17	2001.51	2744.60	4625.35	843.71	6.526	87.01	53.66	15.58	32.38
ITC0245	17.57	4.570	27.90	596.99	475.64	918.12	186.83	885.70	784.93	220.00	263.32	4.234	34.33	30.68	9.548	23.00
ITC0245	33.22	10.59	40.72	3232.65	455.30	1301.25	172.80	7014.64	2146.04	197.34	335.38	4.500	55.96	60.01	13.09	41.08
ITC0245	24.60	4.072	57.54	1146.44	440.54	950.17	165.39	430.86	1194.08	68.92	94.19	4.552	50.29	69.03	11.71	22.99
ITC0249	54.87	24.46	162.87	7935.70	460.11	3155.03	283.78	5884.62	479.84	4.155	61.67	0.744	78.04	54.61	12.14	67.17
ITC0249	27.84	117.58	55.94	5799.83	233.92	1879.48	124.39	6343.88	524.21	12.06	272.70	3.290	78.81	83.67	18.59	53.01
ITC0249	30.16	129.95	72.08	5033.93	341.13	1942.92	144.12	5955.83	571.86	8.789	292.65	3.531	86.13	60.75	13.50	61.14
ITC0250	20.12	9.516	44.74	1904.91	766.55	2138.31	372.92	5704.04	540.82	22.73	556.63	3.039	91.64	72.37	13.07	37.26
ITC0250	12.88	8.318	3.685	4441.37	293.66	1792.79	169.16	2446.92	679.68	19.33	481.90	4.496	59.50	47.82	23.31	31.22
ITC0250	16.51	8.393	3.866	4732.67	318.39	1893.83	129.45	3297.58	783.08	20.20	513.34	4.310	64.42	65.89	19.14	39.79
ITC0250	21.88	8.283	31.17	443.41	576.71	939.42	180.47	5385.88	1015.21	8.483	252.97	3.063	69.41	114.34	17.57	51.27
ITC0250	21.16	15.44	6.709	4185.99	312.65	2000.73	156.00	4908.84	1143.24	32.66	753.64	5.856	84.96	95.17	53.10	65.29
ITC0253	26.29	4.860	24.41	1996.16	129.55	612.05	47.74	1668.56	1677.24	54.94	879.66	3.252	62.35	107.39	13.85	102.30
ITC0253	23.12	8.373	50.56	3322.47	229.30	1123.55	71.63	3549.74	1279.94	66.25	1044.93	5.346	100.74	140.49	13.32	120.66
ITC0253	21.05	6.638	44.73	3265.67	199.01	985.52	72.32	2724.95	1149.85	56.19	846.52	3.989	81.31	85.89	10.97	146.08
ITC0277	32.59	4.212	101.44	3649.20	412.01	1680.29	270.88	7555.98	7113.66	68.46	286.97	6.391	84.13	198.23	36.11	32.94
ITC0277	25.09	3.876	105.32	3352.45	249.08	1095.47	82.49	5180.57	4623.72	63.12	211.51	4.475	73.97	139.49	20.34	21.14
ITC0277	25.17	3.055	78.09	4837.21	297.58	1412.36	87.12	5730.93	6165.58	5.734	83.91	2.345	47.00	14.74	6.747	15.25
ITC0312	38.73	11.18	111.04	1043.57	279.52	629.40	87.98	12932.78	8047.74	3173.15	487.76	22.99	71.71	29.81	25.84	57.38
ITC0312	28.36	7.611	96.23	7341.68	312.91	2134.38	272.42	13288.76	6458.67	2758.54	389.29	14.18	50.83	29.71	20.68	37.61
ITC0312	39.38	5.937	80.63	6082.56	306.24	1833.50	235.55	15216.05	8380.83	2288.48	284.26	9.833	52.93	43.42	19.14	52.11
ITC0361	30.16	16.94	88.95	6332.06	344.55	1866.17	136.62	3630.98	2236.86	1366.26	1160.40	8.373	77.53	137.12	22.61	49.55
ITC0361	33.05	9.190	103.50	10344.13	517.27	3358.02	203.21	3773.56	1965.75	1032.28	976.96	6.563	54.26	112.23	8.612	54.31
ITC0361	18.61	18.88	30.71	5744.54	294.21	1799.90	119.16	3060.36	2304.60	1449.72	1206.24	8.826	83.34	143.34	26.10	30.51
ITC0472	13.31	3.398	2.272	4644.72	308.63	2235.51	202.89	40.15	78.82	31.45	46.33	2.775	157.91	32.47	5.952	17.84
ITC0472	27.77	19.52	33.40	7864.36	332.02	3199.39	171.53	1383.34	794.18	787.48	724.23	4.414	44.41	63.94	8.979	27.00
ITC0472	42.09	24.97	49.68	8232.59	473.71	3716.67	336.50	1316.78	1512.53	1020.66	877.41	5.039	57.95	33.56	7.722	34.88
ITC0587	20.06	8.144	71.34	3667.69	256.00	1338.86	102.88	2174.66	1275.70	1180.11	424.86	4.546	66.80	56.62	8.864	33.11
ITC0587	25.93	6.548	89.55	5330.42	333.15	1835.51	145.74	2641.55	2276.54	1805.57	621.49	4.668	56.46	62.15	12.60	66.13
ITC0587	58.40	11.17	294.31	4080.33	297.93	1412.58	111.66	3116.67	3783.67	3213.82	926.93	7.708	101.82	134.89	19.56	90.14
ITC0649	23.03	8.870	99.01	3873.19	328.34	1370.62	157.41	2577.78	497.57	608.02	651.44	5.720	85.72	100.14	19.02	20.25
ITC0649	18.49	11.26	43.35	3419.10	256.40	1261.00	113.52	766.93	470.41	524.46	588.80	6.298	62.30	29.31	8.636	18.71
ITC0649	8.269	6.413	33.72	3339.84	298.86	1166.12	92.37	1019.36	433.37	440.98	516.53	4.543	57.57	57.32	8.411	27.12
ITC0653	24.73	3.581	58.00	2895.31	233.44	960.56	88.88	950.87	1798.76	11.00	40.52	8.232	89.06	60.79	11.33	52.62
ITC0653	18.11	2.438	50.27	4234.34	337.14	1539.55	146.25	977.05	1443.58	9.562	32.34	5.078	63.13	60.59	9.070	34.49
ITC0653	34.33	2.793	48.60	4581.73	467.27	1551.37	193.03	1088.78	2334.08	9.125	29.59	5.707	92.73	88.84	10.49	72.92
ITC0654	21.50	4.232	42.21	4149.46	349.43	1712.41	153.25	2223.49	1695.89	35.79	334.58	3.999	57.03	22.17	11.28	45.82
ITC0654	28.77	5.471	148.90	4048.26	291.27	1832.42	168.84	4192.32	2147.30	59.72	292.27	7.224	97.56	39.18	57.98	59.41
ITC0654	31.43	4.219	88.02	5437.75	377.09	2169.33	189.15	1889.78	2111.20	49.32	222.20	4.509	58.99	43.67	17.06	44.87
ITC0659	16.53	6.868	3.194	2701.78	200.41	1018.78	86.81	2890.61	1300.68	320.60	425.77	4.653	58.13	115.14	23.74	27.96
ITC0659	24.98	7.221	3.742	5032.91	321.49	1869.32	160.46	3103.33	1322.18	384.22	487.66	4.409	70.19	84.99	27.98	35.52
ITC0659	20.87	10.08	3.771	4255.02	316.16	1577.72	155.39	3299.48	799.12	503.14	615.93	5.365	59.78	85.63	40.47	28.08
ITC0662	15.27	2.068	33.52	4625.26	202.58	1871.60	129.06	921.52	1079.22	6.959	337.13	3.041	37.00	15.75	7.813	24.05
ITC0662	22.95	2.382	24.72	4402.24	300.50	1513.33	144.12	2129.80	1848.81	6.951	357.19	4.294	67.65	45.38	7.621	39.65
ITC0662	22.13	3.030	11.93	5985.22	406.01	1948.32	136.91	867.16	1764.48	7.233	351.10	4.419	67.65	36.31	8.409	89.25
ITC0767	29.58	32.63	84.12	4266.43	579.47	1807.02	277.98	1461.99	328.31	1796.44	710.55	4.335	61.70	34.85	14.07	25.26
ITC0767	20.05	22.43	5.494	4625.83	367.19	1662.72	104.59	1603.69	1550.63	1566.12	539.20	6.214	79.42	47.69	10.78	38.50
ITC0767	26.47	13.58	55.08	4234.59	320.32	1399.70	139.39	530.94	467.12	1191.63	633.72	11.21	76.14	70.68	14.60	76.63
ITC0767	28.96	28.82	78.32	5428.71	217.30	1575.48	127.00	1275.86	439.37	1675.53	695.19	9.836	73.25	63.52	14.49	66.36
ITC0767	25.52	20.44	3.737	5765.99	372.92	2348.19	186.77	1341.48	1732.05	1728.30	547.76	5.235	71.20	51.06	7.166	53.17
ITC0767	26.28	21.69	3.866	6088.99	464.53	2327.61	200.03	1804.66	1528.05	1585.71	525.71	5.240	68.74	45.64	8.423	35.14
ITC0769	13.45	5.523	43.23	4833.92	310.16	1720.60	136.65	1331.29	1075.15	586.52	550.87	3.860	55.89	66.59	8.276	29.65
ITC0769	18.51	5.800	58.06	4127.69	267.49	1523.36	103.08	1109.49	1101.36	606.20	610.96	5.108	80.89	44.24	9.081	55.27
ITC0769	8.202	5.825	37.70	5001.81	260.21	1461.19	80.98	1250.11	400.89	492.78	540.38	4.375	70.55	50.72	6.772	69.62
ITC1120	21.27	9.408	39.77	1643.27	245.11	706.84	126.34	867.16	371.64	34.23	707.79	7.201	100.75	29.79	9.090	28.68
ITC1120	48.05	15.88	79.11	2206.35	307.48	901.61	143.86	1144.50	336.40	19.76	563.12	8.393	81.53	38.96	43.61	54.71
ITC1120	31.40	10.79	75.67	5507.15	177.12	1535.02	90.59	1686.41	284.69	24.11	751.87	6.592	56.94	29.11	12.95	44.34
ITC1121	21.25	7.532	60.64	7066.93	325.50	2034.40	151.32	1567.03	1256.70	19.51	455.09	2.806	35.39	22.71	6.220	30.66
ITC1121	22.64	11.30	65.38	6587.79	326.77	2020.61	146.41	2363.18	1519.64	13.28	385.05	2.648	35.53	22.77	9.181	24.52
ITC1121	26.21	6.460	77.60	5683.27	203.10	1671.14	115.71	5320.87	2559.53	15.78	310.25	4.624	70.38	29.49	9.067	37.70
ITC1122	32.73	14.24	112.25	1868.78	380.46	992.44	138.04	2337.73	8126.37	23.24	1000.80	14.17	97.36	80.58	26.95	27.04
ITC1122	34.76	14.94	124.55	3121.36	281.96	1106.35	96.31	1984.71	7704.24	24.47	1034.46	6.109	77.86	40.06	13.28	15.11
ITC1122	24.61	11.45	63.02	7250.10	319.66	2361.33	118.99	2425.94	8231.85	18.29	866.07	16.19	102.24	90.71	30.37	30.02
ITC1177	6.261	4.762	15.32	4003.30	508.01	2106.99	367.08	1515.71	205.59	13.91	561.25	4.018	48.38	35.04	13.68	26.35
ITC1177	7.137	6.071	15.70	4637.01	441.27	2391.49	355.50	1853.53	217.47	15.44	692.57	6.554	58.70	39.42	9.021	33.65
ITC1177	8.854	5.958	12.07	3566.73	690.98	2447.04	435.48	2926.70	5192.77	28.41	900.47	4.160	60.31	37.71	15.68	27.01
ITC1177	9.821	6.722	15.00	2460.58	658.74	1751.66	293.40	5268.52	5250.79	14.08	804.38	14.20	62.82	52.08	13.17	45.26
ITC1177	18.01	7.289	30.68	7874.17	349.49	2897.89	149.51	1820.62	4135.36	19.53	649.37	7.982	38.16	19.00	12.35	25.95
ITC1177	10.03	10.56	11.16	8412.61	792.56	3398.70	307.75	3991.57	4068.62	15.10	603.53	10.37	48.41	59.43	14.84	54.30
ITC1177	9.262	6.058	30.54	3630.78	372.32	1928.75	279.77	2735.66	304.97	19.42	880.36	6.279	65.20	58.12	10.51	42.45
ITC1187	44.62	13.12	134.71	6249.06	198.04	1918.72	104.69	1261.73	6255.46	18.17	441.90	5.331	107.57	61.29	13.62	45.02
ITC1187	31.51	13.18	93.38	5382.11	181.76	1570.06	93.74	823.08	8406.98	20.55	515.04	6.214	131.12	102.15	22.70	54.95
ITC1187	39.92	13.16	119.89	10332.48	245.26	3229.07	155.04	1104.44	7635.49	19.70	488.82	5.897	122.67	87.50	19.44	51.39
ITC1325	29.76	10.87	63.38	8388.04	293.61	2404.09	237.55	4185.44	6033.75	339.83	577.29	4.524	70.88	103.56	15.89	74.25
ITC1325	29.32	9.868	134.95	5257.53	386.65	1649.94	130.21	2373.89	1298.92	323.12	541.61	4.918	72.85	68.03	11.32	53.00
ITC1325	41.77	8.850	145.62	4015.25	260.31	1192.75	81.10	3550.88	1904.30	239.00	463.02	4.902	99.16	154.32	13.75	70.77
ITC1441	5.339	8.478	2.993	3180.27	257.19	1100.25	105.22	819.11	2200.40	31.37	556.87	2.958	47.14	66.32	12.28	18.63
ITC1441	9.243	6.998	10.18	3138.28	171.84	968.18	57.85	1000.78	4607.47	60.47	803.36	5.800	129.11	44.68	11.28	35.29
ITC1441	10.33	5.556	29.01	2278.55	223.09	1112.85	90.57	993.23	221.27	32.72	481.28	5.757	70.15	25.92	6.254	20.44
ITC1441	11.17	7.529	8.383	4700.22	303.49	1470.63	101.98	2312.39	4041.70	42.68	691.11	4.768	101.99	37.50	10.73	25.91
ITC1441	25.03	7.250	20.54	4402.52	254.18	1546.14	112.21	1652.92	180.05	30.52	511.41	5.780	88.22	33.69	8.959	30.63
ITC1441	6.211	8.517	4.448	4283.56	207.86	1411.37	65.90	820.21	2706.73	34.67	616.37	3.348	60.60	76.92	11.40	19.71
ITC1441	14.51	6.298	21.41	4683.37	288.05	1607.52	113.52	1666.73	68.98	47.75	654.14	5.822	75.85	122.23	22.30	31.87
ITC1483	56.52	7.194	253.75	8637.45	364.25	2584.61	160.16	927.80	3443.77	4983.85	690.77	6.819	66.56	32.38	17.58	54.88
ITC1483	35.37	16.15	107.59	8079.04	231.29	2414.31	138.05	855.80	3715.36	4808.72	590.30	6.477	49.69	25.39	19.46	35.39
ITC1483	27.54	15.91	37.06	6001.06	305.28	2058.03	129.92	1157.16	2175.11	2446.65	524.25	5.295	65.29	25.87	12.91	34.04
ITC1483	36.77	15.69	25.10	6255.92	380.22	2393.15	150.83	1158.53	2458.25	2835.01	599.40	4.216	74.32	27.18	7.311	52.60
ITC1483	48.12	13.25	193.35	9059.72	230.69	2958.46	164.41	1164.44	4332.90	5521.26	756.07	6.644	88.27	40.22	12.78	40.87
ITC1483	21.02	10.60	53.12	6803.45	537.86	2691.88	247.49	525.07	1801.60	2271.42	309.28	5.931	70.10	41.08	15.32	46.69
ITC1483	51.13	26.78	89.51	6319.75	308.27	2509.57	188.76	2077.25	3479.48	5028.13	1002.28	9.808	106.38	47.03	31.14	63.75

Variety	Guanine	Histidine	Inositol-myo	Isocitric acid	kaempferol-3-O-rutinoside	Kaempferol-rutinoside	Lutein	Malic acid	m-Coumaric acid	Neochlorogenic acid	Neoxanthin	o-Coumaric acid	p-Coumaric acid	Phenylalanine	Pheophytin a	Phylloquinone
ITC0084	2.111	2.146	110.20	4064.73	28.31	433.41	535.56	1528.38	3.065	1021.39	171.39	2.067	10.58	48.14	447.47	17.06
ITC0084	4.007	2.325	146.03	1461.20	40.43	479.56	499.96	1801.76	4.988	1971.49	187.93	3.695	11.05	40.37	529.11	15.09
ITC0084	4.473	2.769	130.95	2682.52	18.93	515.81	585.62	1500.30	6.136	1485.54	217.50	5.589	15.94	65.50	805.79	18.41
ITC0084	3.609	2.287	120.73	2725.72	14.96	428.95	539.01	1254.43	5.864	1209.37	174.60	6.338	12.35	46.00	406.19	16.54
ITC0084	1.123	2.004	83.85	2551.18	17.95	334.85	718.17	957.15	4.783	1237.61	227.59	4.354	8.532	36.08	839.77	20.09
ITC0084	39.68	2.237	136.45	2691.44	31.71	463.38	767.83	1459.09	6.055	2256.80	231.85	3.562	11.10	46.46	539.04	23.65
ITC0111	3.686	2.637	104.89	820.04	17.40	653.52	593.54	797.62	6.764	736.33	192.48	3.157	8.202	22.97	272.76	20.23
ITC0111	3.885	4.441	172.28	2021.81	18.21	905.38	672.22	775.17	4.353	802.23	206.25	2.093	8.278	48.12	515.29	23.01
ITC0111	3.333	2.513	118.15	926.37	15.53	841.67	702.71	630.55	6.815	880.61	214.67	2.477	8.629	32.74	439.78	24.91
ITC0112	3.700	3.212	170.74	1781.78	31.56	995.09	888.24	924.80	5.833	1806.06	259.87	2.696	9.217	38.90	588.77	27.64
ITC0112	5.102	3.438	204.91	741.07	32.20	959.09	684.94	936.38	12.02	1396.41	215.46	4.939	11.47	48.65	472.87	22.11
ITC0112	4.822	3.392	198.08	1573.72	32.07	987.89	709.89	934.06	10.78	1724.16	238.06	4.491	11.02	46.70	879.65	22.38
ITC0121	4.444	7.037	162.00	693.15	8.939	672.94	466.72	639.50	7.951	1195.80	163.27	4.916	9.606	32.49	493.50	20.41
ITC0121	1.985	2.675	104.13	812.47	4.980	538.97	372.49	406.77	3.866	1453.17	131.34	2.710	15.32	14.85	746.50	15.60
ITC0121	3.327	1.598	63.94	142.25	9.147	324.26	436.86	284.08	2.058	1061.16	181.86	1.368	12.88	10.74	520.34	18.64
ITC0121	4.220	1.632	148.56	2163.04	6.206	698.08	437.19	856.82	4.399	1834.09	162.31	2.648	7.264	28.66	356.49	18.09
ITC0121	4.997	4.224	156.87	1814.53	10.16	686.21	623.04	835.81	4.488	1274.93	187.39	1.896	6.474	32.89	548.60	22.52
ITC0123	1.271	3.030	105.68	1231.54	5.787	983.77	369.39	685.96	4.988	383.08	177.70	4.652	24.43	33.09	285.53	14.35
ITC0123	0.531	1.960	107.18	1099.05	10.44	1041.97	397.28	645.66	5.165	661.18	203.52	3.559	33.37	39.64	278.38	15.58
ITC0123	1.123	2.816	106.88	1205.05	9.513	1030.33	391.70	677.90	5.129	605.58	198.36	4.434	31.58	38.33	284.10	15.33
ITC0245	0.829	2.045	51.35	462.03	4.130	199.95	434.10	328.47	4.531	392.08	140.72	5.372	14.22	26.53	861.85	19.53
ITC0245	2.185	2.962	97.80	1662.25	5.946	296.05	540.35	1288.85	8.580	256.63	158.67	6.325	18.43	48.04	615.63	21.32
ITC0245	4.554	1.430	110.58	143.26	6.135	144.44	624.38	558.42	8.592	355.35	195.49	9.241	14.67	17.80	785.80	24.93
ITC0249	4.222	1.271	183.97	7938.71	285.45	215.71	786.74	1731.74	13.10	1818.17	245.54	9.147	21.02	53.44	1691.87	28.26
ITC0249	3.333	2.184	162.22	8810.19	251.44	239.13	534.75	1665.43	10.00	712.13	187.64	6.983	16.05	57.89	963.57	22.15
ITC0249	3.843	2.342	165.08	8313.06	284.37	257.06	584.01	1236.02	11.60	809.75	202.00	8.100	18.61	52.15	919.83	23.66
ITC0250	4.234	2.831	119.76	1656.70	252.42	228.41	813.59	528.20	5.599	309.14	203.77	4.257	9.376	39.96	3215.32	21.78
ITC0250	2.769	1.997	87.07	1557.79	283.50	193.88	658.58	590.15	2.789	183.10	239.67	7.969	15.13	38.87	797.54	23.38
ITC0250	3.007	2.529	112.33	1027.70	335.18	232.16	673.54	644.24	3.111	185.49	232.56	6.443	12.30	43.50	848.78	24.48
ITC0250	6.673	3.294	129.38	3367.17	235.83	281.97	478.79	845.40	8.982	353.58	142.53	6.798	15.91	66.66	1339.45	17.27
ITC0250	4.084	2.293	155.10	1835.49	453.17	324.27	601.71	899.30	3.989	262.47	209.41	8.884	19.77	60.65	1075.55	22.28
ITC0253	1.583	2.174	135.01	1522.26	249.24	152.81	300.96	594.04	4.459	835.16	118.17	3.180	22.05	22.35	236.38	15.97
ITC0253	3.121	2.526	146.82	2426.09	258.05	181.90	379.55	1088.31	4.255	1150.72	151.03	5.265	19.95	28.79	368.18	16.10
ITC0253	2.161	3.165	146.22	1813.91	236.91	171.30	351.08	570.23	4.033	608.73	144.48	6.719	19.50	26.74	335.58	17.35
ITC0277	3.979	4.163	209.01	4273.13	30.79	410.15	571.37	2133.66	7.200	672.95	158.40	5.932	14.57	46.90	1449.98	22.19
ITC0277	4.072	2.866	161.50	1570.19	17.92	430.08	364.38	1220.33	5.002	568.77	165.71	4.653	11.05	20.48	182.79	18.02
ITC0277	1.235	1.092	45.53	1526.48	22.66	355.49	481.36	1174.91	5.321	858.16	179.22	2.977	7.927	18.64	271.13	26.18
ITC0312	1.938	7.740	86.41	4182.40	56.30	309.21	408.81	2570.10	5.679	1452.34	122.77	8.741	12.36	84.22	406.28	15.70
ITC0312	1.826	6.597	76.30	1281.77	23.65	279.38	636.99	1708.70	4.717	1410.34	189.57	3.768	8.663	76.08	1308.37	26.48
ITC0312	2.217	8.615	88.05	411.12	36.87	243.34	591.38	1758.50	3.939	2790.50	176.22	2.310	7.999	144.51	1128.02	24.32
ITC0361	24.86	1.999	167.76	2879.18	14.38	607.50	684.46	1931.23	7.957	804.55	221.35	16.77	26.04	63.53	523.22	23.53
ITC0361	7.099	1.436	147.56	2667.01	9.148	490.69	917.35	1616.21	4.690	899.18	274.89	12.81	16.78	42.94	907.90	28.63
ITC0361	29.30	2.140	172.81	2932.19	15.69	636.69	588.12	2009.94	8.774	425.82	186.67	17.75	28.35	68.67	388.20	20.83
ITC0472	2.212	0.804	38.08	5.550	11.68	66.92	521.06	1.428	5.067	14.24	189.56	4.021	11.02	3.237	828.49	17.26
ITC0472	1.345	4.039	91.05	598.03	68.92	535.75	651.84	578.83	6.176	156.51	256.65	3.526	6.342	58.42	467.80	20.76
ITC0472	1.695	3.044	91.87	314.20	88.61	449.26	765.36	485.56	7.958	468.60	267.98	3.650	7.578	61.19	1156.14	20.95
ITC0587	2.719	1.700	83.98	2806.70	13.96	240.27	418.35	1068.83	5.044	1054.83	179.00	3.840	8.333	27.72	526.10	20.87
ITC0587	3.136	2.081	111.81	2880.43	39.52	309.34	631.33	1138.52	8.260	1149.39	203.71	2.707	8.077	37.88	602.89	27.87
ITC0587	6.385	4.889	209.01	2501.82	44.76	535.04	528.18	1127.88	11.37	1943.53	195.30	5.811	18.40	80.04	641.53	25.83
ITC0649	4.162	1.916	135.92	662.94	24.91	630.24	534.11	773.51	9.263	261.94	187.21	6.838	11.79	42.20	500.50	18.52
ITC0649	2.302	1.634	85.56	471.79	18.20	452.58	489.91	518.49	9.149	165.21	157.00	8.536	12.66	33.53	511.01	18.39
ITC0649	1.819	1.363	83.22	939.26	16.65	380.72	417.07	610.26	4.209	201.00	151.83	4.566	7.519	27.31	383.54	16.34
ITC0653	2.747	3.844	146.59	1221.17	51.23	517.27	421.58	675.50	7.238	638.78	142.42	12.17	23.73	33.69	482.08	18.65
ITC0653	2.589	3.276	129.43	374.25	21.52	467.36	530.49	449.10	6.012	620.31	166.55	5.244	16.63	30.43	645.30	21.11
ITC0653	3.334	5.020	169.15	391.69	79.87	450.54	555.74	695.19	6.044	1263.89	173.14	7.458	21.91	63.99	724.22	21.17
ITC0654	2.966	3.250	80.52	2478.12	118.46	444.97	489.83	884.85	3.705	576.22	157.98	2.138	13.16	52.83	849.68	18.31
ITC0654	6.674	6.668	122.28	1025.95	196.14	662.89	566.38	1305.70	5.283	654.30	172.55	4.540	19.70	77.24	1042.83	18.82
ITC0654	4.818	2.915	91.38	778.93	158.17	371.93	646.26	684.68	5.309	546.04	187.99	4.373	19.41	57.49	1072.92	23.78
ITC0659	2.301	2.041	140.15	871.73	132.19	486.43	383.78	649.55	6.839	514.94	168.80	2.282	6.411	42.87	455.25	15.14
ITC0659	1.676	2.331	113.60	1673.26	175.09	409.21	628.86	897.81	8.224	545.81	235.14	3.718	7.932	42.27	728.94	21.72
ITC0659	1.510	3.589	100.84	1841.34	148.87	441.67	547.22	969.16	9.030	249.06	226.34	4.182	7.587	44.67	579.44	19.56
ITC0662	2.128	1.638	53.30	389.66	44.14	72.80	568.77	344.69	4.003	493.76	174.00	3.535	8.894	29.18	639.34	20.75
ITC0662	3.183	3.281	131.63	515.84	53.88	180.94	553.79	397.33	2.998	1039.50	161.04	2.547	8.426	25.49	529.43	25.97
ITC0662	2.279	2.993	109.66	728.42	67.25	146.47	598.29	250.24	2.279	1375.49	164.86	1.675	5.141	20.30	374.27	27.56
ITC0767	0.880	0.995	73.81	839.89	13.11	490.74	775.57	769.54	4.580	1359.94	224.04	4.863	27.36	50.44	868.17	19.91
ITC0767	3.052	1.570	93.82	650.41	9.558	523.54	610.46	614.63	7.209	1294.60	211.40	4.578	9.319	41.45	372.50	19.30
ITC0767	6.277	3.908	128.49	193.74	11.76	753.65	679.14	510.09	6.381	536.13	195.98	3.820	27.94	34.78	151.70	21.32
ITC0767	5.198	3.325	117.56	710.71	12.84	701.09	617.78	717.67	6.021	1195.25	222.52	4.655	27.82	47.31	292.27	21.20
ITC0767	1.659	1.855	100.76	1423.22	10.71	584.61	796.01	724.19	5.115	1342.31	257.61	2.238	8.757	36.49	899.50	25.74
ITC0767	2.609	2.452	95.46	551.95	10.12	520.62	897.69	644.62	6.398	1282.04	258.88	2.770	7.589	38.83	897.74	26.11
ITC0769	2.523	1.361	86.15	800.55	23.70	401.54	598.84	533.17	4.976	244.48	192.02	3.654	8.810	25.92	654.11	23.90
ITC0769	3.010	2.055	110.58	947.17	29.97	439.72	489.84	618.98	4.123	414.05	169.64	2.709	10.28	28.93	644.46	18.96
ITC0769	2.444	2.289	94.83	1059.51	23.73	419.07	460.28	679.57	1.943	154.97	169.53	2.543	5.510	22.22	220.04	19.70
ITC1120	3.267	1.929	122.16	597.05	20.47	851.89	379.36	625.81	5.808	363.70	137.98	5.563	24.68	27.56	471.10	14.33
ITC1120	0.727	3.488	136.76	599.74	35.32	807.64	464.10	549.51	7.308	1065.91	145.67	3.712	64.79	67.36	528.00	19.21
ITC1120	3.649	1.400	110.31	770.23	34.62	557.70	412.22	507.90	5.591	1572.19	265.76	2.106	45.33	35.43	446.31	18.44
ITC1121	0.521	2.872	75.88	1135.65	142.62	101.11	587.43	335.77	3.937	257.69	225.50	4.248	6.577	24.58	1415.57	25.01
ITC1121	1.076	2.960	78.22	1604.77	155.23	120.05	582.38	455.92	6.174	176.17	233.36	4.644	8.447	38.96	868.85	24.96
ITC1121	1.523	2.816	91.44	1536.41	244.82	181.69	472.98	572.96	4.063	497.24	175.49	5.580	7.681	26.38	805.05	18.53
ITC1122	7.458	3.766	133.36	1235.95	86.89	351.07	409.19	935.37	11.13	778.25	128.44	8.356	14.02	43.77	686.77	15.33
ITC1122	2.211	4.443	110.72	1266.94	82.26	363.02	494.47	581.32	6.596	518.32	163.95	6.910	10.37	42.87	313.46	19.44
ITC1122	8.769	1.058	139.02	1111.92	88.04	303.23	578.70	1023.84	12.26	843.20	196.83	8.717	14.93	43.99	674.59	17.68
ITC1177	1.014	3.388	52.13	532.50	4.843	314.14	602.23	441.40	6.283	41.47	183.07	6.983	21.40	25.59	1302.36	19.72
ITC1177	1.100	3.211	68.62	787.01	6.899	354.11	602.29	556.65	6.433	51.63	198.18	7.938	23.62	33.08	1260.48	19.70
ITC1177	0.721	3.264	67.60	1137.89	4.023	396.62	784.07	481.27	9.537	84.64	244.03	5.810	12.60	31.00	2815.56	21.16
ITC1177	2.080	2.582	101.25	1215.57	7.381	283.38	641.41	699.79	9.561	153.22	217.97	4.204	8.094	31.34	2089.30	19.63
ITC1177	0.864	1.093	43.85	792.36	10.54	246.95	596.77	491.45	11.07	190.19	201.80	6.020	8.667	25.89	780.68	20.59
ITC1177	3.780	1.448	89.10	977.00	7.701	218.87	892.31	653.01	8.194	103.85	253.11	3.777	7.572	31.41	2307.64	30.52
ITC1177	1.795	4.253	96.74	878.71	6.610	432.56	555.07	784.50	9.795	58.13	173.57	8.860	27.81	43.69	1246.17	18.43
ITC1187	2.830	2.572	148.74	430.06	26.81	1058.27	600.62	1118.70	10.22	1623.47	237.52	7.132	16.39	40.26	834.32	20.78
ITC1187	3.455	7.234	206.17	249.41	34.55	2179.65	544.80	839.23	13.98	1188.40	223.66	9.761	22.43	80.19	613.60	17.90
ITC1187	3.231	5.562	185.58	365.28	31.78	1777.55	963.58	1018.49	12.63	1467.46	297.03	8.818	20.26	65.87	1246.15	25.33
ITC1325	1.495	5.110	142.12	1315.32	16.18	663.03	756.81	1268.04	8.806	914.11	255.91	5.516	27.86	57.48	1674.82	32.96
ITC1325	4.429	3.570	118.21	703.04	15.08	654.80	569.44	771.55	6.745	991.64	179.57	3.058	7.201	45.09	344.41	23.57
ITC1325	6.933	2.974	159.79	924.56	23.62	792.84	408.79	1199.64	8.748	1410.86	151.36	3.762	9.119	43.77	217.08	19.78
ITC1441	1.209	1.023	94.61	563.86	6.935	287.93	356.30	581.25	4.695	23.45	157.41	4.144	12.59	27.41	196.46	15.36
ITC1441	2.359	3.709	144.86	637.67	9.322	691.70	343.30	607.39	5.127	104.54	155.37	5.716	18.10	22.05	302.90	15.20
ITC1441	2.044	1.920	89.93	346.21	6.594	469.45	374.90	478.11	5.387	111.35	156.44	4.779	8.364	19.45	664.70	15.48
ITC1441	2.810	4.983	109.06	1142.12	12.53	705.42	441.93	758.61	4.551	64.69	192.00	6.000	14.99	26.64	354.69	20.60
ITC1441	2.564	2.263	95.25	983.64	11.86	476.68	513.46	807.60	5.033	88.49	186.98	7.856	10.80	24.94	504.76	18.61
ITC1441	1.785	1.557	107.89	512.21	6.059	429.62	371.64	474.17	4.824	30.80	158.27	5.663	12.17	17.47	525.57	15.36
ITC1441	2.484	1.958	139.44	638.63	7.413	517.76	536.52	688.72	7.998	35.65	186.33	8.451	13.93	31.56	566.59	20.05
ITC1483	0.579	4.960	148.03	867.72	18.58	500.78	770.49	919.07	4.414	1006.60	282.22	6.622	21.08	77.11	908.30	26.20
ITC1483	0.544	2.043	97.78	708.14	12.20	500.39	738.26	581.08	3.913	475.19	326.63	7.281	27.14	55.91	950.80	22.46
ITC1483	2.558	2.561	137.15	324.37	8.660	634.28	733.59	464.29	6.095	940.57	249.64	3.231	6.928	39.70	545.36	23.23
ITC1483	1.428	2.173	150.11	1257.96	10.81	683.94	635.11	695.72	4.340	1046.41	259.22	1.625	7.534	39.52	422.13	18.79
ITC1483	0.624	1.302	157.33	833.05	11.27	890.51	684.28	658.62	3.669	1142.39	346.21	5.968	28.72	46.23	885.57	20.49
ITC1483	2.868	2.620	109.40	173.76	11.91	592.65	767.26	375.04	5.121	207.64	278.20	2.382	16.63	25.31	1466.26	22.11
ITC1483	4.321	3.660	195.46	548.16	16.01	841.65	726.78	739.57	10.29	1263.16	284.15	6.127	14.03	79.77	686.81	20.60

Variety	Phytoene	Proline	Quercetin	Quercetin-glucoside	Quinic acid	Quinone	Rutin	RutinII	Sedoheptulose	Shikimic acid	Sucrose	Tryptophan	Violaxanthin isomer1	Violaxanthin isomer2	Violaxanthin 13-cis	Violaxanthin 9-cis
ITC0084	26.91	1.085	3.202	619.60	952.37	35.85	87.77	619.23	26.71	64.38	86.05	44.39	95.02	146.46	101.44	93.48
ITC0084	23.19	2.498	10.75	664.21	1490.71	38.06	155.63	719.85	38.94	119.14	117.33	30.71	94.31	158.17	103.28	93.12
ITC0084	23.10	2.066	18.24	734.16	1334.58	37.34	110.57	728.52	49.69	83.50	110.02	65.00	95.14	168.33	107.04	93.77
ITC0084	22.22	1.269	9.845	844.91	1115.63	47.48	49.04	702.33	24.41	251.92	97.70	58.98	103.75	183.03	103.96	96.04
ITC0084	21.86	0.934	14.67	806.35	998.67	45.57	51.71	592.82	30.91	200.96	82.66	56.44	91.70	200.33	106.51	88.35
ITC0084	29.39	1.727	10.58	1046.60	1617.20	45.94	65.56	844.44	32.99	256.68	127.72	32.11	95.75	197.09	110.77	93.68
ITC0111	25.55	1.023	5.412	308.40	88.15	56.90	23.23	774.16	16.77	57.48	148.74	19.88	105.84	191.34	107.84	96.28
ITC0111	26.48	2.517	4.579	659.09	41.20	63.32	20.45	954.71	29.69	78.49	165.20	48.26	100.51	215.90	106.47	94.83
ITC0111	28.40	1.397	6.030	581.68	887.06	53.66	26.51	907.83	25.32	53.47	143.06	32.83	99.11	236.38	110.62	94.97
ITC0112	28.09	1.408	5.060	833.95	22.44	36.46	28.32	1027.39	27.25	75.13	178.20	55.18	107.03	272.87	116.16	97.54
ITC0112	25.01	1.088	7.357	662.28	35.27	62.98	27.41	977.98	27.37	89.76	204.18	46.35	105.53	222.46	112.87	96.46
ITC0112	23.69	1.344	6.898	799.63	32.71	66.57	28.14	1017.51	27.35	86.84	198.99	53.41	103.31	246.47	112.70	94.67
ITC0121	26.45	2.501	6.577	415.39	50.90	43.99	24.70	955.60	35.68	67.56	184.54	33.72	100.99	180.85	102.48	94.72
ITC0121	21.55	1.357	2.158	550.82	29.89	57.15	15.59	727.97	25.48	28.85	162.28	15.46	95.77	131.00	92.85	89.41
ITC0121	23.80	0.445	1.029	342.95	10.53	48.44	24.23	373.88	9.069	5.392	78.53	27.16	95.21	181.57	99.32	90.67
ITC0121	24.99	2.321	4.247	654.46	44.19	36.05	12.53	843.44	26.82	116.30	177.23	18.13	100.27	172.33	100.46	93.97
ITC0121	26.01	1.497	3.162	703.97	61.97	65.31	20.43	859.69	26.11	70.42	150.59	28.73	96.59	227.93	105.79	93.87
ITC0123	22.68	1.148	2.681	86.13	137.13	46.83	12.79	688.75	34.25	30.01	180.99	20.25	89.42	154.24	93.25	85.89
ITC0123	22.17	1.396	4.809	187.92	124.63	49.32	15.80	843.91	38.76	29.56	196.00	41.71	93.48	175.65	98.73	90.61
ITC0123	22.58	1.346	4.384	167.57	134.63	48.82	15.20	812.89	37.85	29.92	193.00	37.42	92.67	171.37	97.63	89.66
ITC0245	22.78	0.773	16.94	328.01	65.74	108.34	33.57	349.90	14.61	31.73	67.38	40.67	97.65	152.83	97.52	91.22
ITC0245	29.10	1.102	31.40	661.91	105.43	66.93	32.92	572.33	33.08	34.01	114.66	108.44	95.23	130.40	98.50	91.62
ITC0245	29.57	0.935	3.346	325.70	164.04	55.31	26.79	418.95	11.79	43.08	99.17	25.82	87.05	145.53	102.42	88.53
ITC0249	21.01	1.272	15.90	826.44	415.35	84.52	444.28	313.25	24.10	76.81	163.27	70.98	95.83	260.98	112.82	91.71
ITC0249	21.69	1.378	15.26	792.98	393.95	85.68	422.17	359.00	15.58	85.88	165.21	57.80	93.00	200.08	101.52	87.83
ITC0249	21.36	1.242	15.31	795.72	274.47	87.53	475.74	296.67	33.72	32.33	162.06	70.97	92.40	184.17	104.26	88.66
ITC0250	22.28	1.383	7.220	763.56	96.11	61.49	467.28	328.05	19.28	27.20	182.20	56.01	104.30	155.36	111.96	99.72
ITC0250	23.11	1.684	8.976	368.35	79.77	57.83	367.20	255.59	20.36	51.11	126.40	71.97	96.47	183.25	109.06	96.02
ITC0250	23.01	1.328	7.707	466.48	86.61	49.39	432.83	291.38	21.18	50.67	129.10	72.80	99.38	182.44	108.80	95.82
ITC0250	20.65	1.231	6.042	404.60	60.70	90.36	447.90	396.10	37.00	31.37	145.15	86.67	93.21	136.99	97.90	89.39
ITC0250	22.49	1.303	12.05	635.27	131.06	70.12	609.56	396.24	51.23	68.66	159.46	110.66	100.31	180.24	108.56	95.77
ITC0253	25.19	1.376	3.355	721.20	185.93	14.51	558.90	366.94	34.89	41.38	132.32	26.94	93.83	114.05	93.95	91.23
ITC0253	22.55	3.197	17.04	785.02	105.75	47.41	651.06	378.59	41.75	127.07	214.69	20.78	101.64	140.25	98.53	94.28
ITC0253	23.66	1.686	23.01	650.88	176.31	14.81	524.10	330.23	31.00	68.50	174.95	21.57	96.07	136.78	97.48	92.20
ITC0277	21.89	1.423	5.582	726.72	1048.11	68.67	107.12	707.11	38.74	213.09	158.85	45.53	101.29	153.07	101.68	95.42
ITC0277	20.30	1.236	8.861	896.29	486.66	44.47	67.50	731.88	19.72	116.49	168.88	32.54	90.74	170.62	95.19	87.85
ITC0277	21.93	0.341	6.102	481.24	74.96	46.23	61.32	466.78	15.75	18.64	99.31	20.40	99.43	212.91	98.17	93.26
ITC0312	27.33	1.719	8.212	505.83	887.27	108.20	158.64	526.70	38.75	70.00	145.25	80.97	94.57	131.05	96.98	89.02
ITC0312	29.94	1.125	4.788	610.23	294.15	52.54	118.98	482.55	26.05	37.03	96.82	104.05	97.73	199.93	101.97	93.63
ITC0312	29.42	1.297	2.764	933.44	225.37	97.07	157.41	444.82	27.42	23.47	90.62	298.67	97.10	186.16	100.97	92.71
ITC0361	36.67	1.644	8.721	284.18	153.75	58.69	22.13	562.96	32.15	46.70	148.10	38.79	103.74	204.41	110.17	101.76
ITC0361	37.05	1.661	7.960	256.57	87.39	68.56	17.38	448.26	30.06	33.66	114.87	27.34	116.55	236.46	117.21	109.25
ITC0361	41.21	1.577	8.911	291.07	170.34	51.55	23.32	591.62	32.67	49.96	156.40	41.65	101.11	194.49	100.75	93.89
ITC0472	22.45	1.850	8.526	43.75	6.274	48.13	17.37	133.72	3.076	4.605	372.02	2.031	100.48	163.70	106.35	95.46
ITC0472	21.84	1.049	3.229	33.61	88.73	24.35	13.94	350.25	17.81	51.55	100.05	51.37	105.86	204.50	109.18	96.25
ITC0472	21.84	1.092	2.749	61.23	40.68	70.56	33.40	326.09	21.32	30.20	121.80	58.24	107.92	235.22	114.99	97.22
ITC0587	22.44	1.407	5.536	161.79	70.22	42.23	152.94	484.37	19.22	142.12	134.12	21.08	100.64	149.88	101.06	94.90
ITC0587	25.78	1.165	3.639	361.81	158.86	62.30	190.16	595.62	27.93	49.58	114.08	23.33	98.25	175.91	106.92	97.05
ITC0587	23.67	2.855	10.91	645.40	127.65	51.01	305.41	962.11	69.73	113.47	204.66	59.05	96.98	147.03	103.42	95.52
ITC0649	24.70	2.458	5.387	373.77	212.11	53.95	48.12	827.79	36.61	44.46	178.52	52.78	97.24	152.32	103.95	95.72
ITC0649	25.42	1.149	7.015	288.61	87.41	40.51	44.64	520.87	26.94	48.49	137.72	17.14	98.29	148.84	101.58	92.97
ITC0649	25.11	0.837	2.294	409.33	102.63	41.97	47.81	460.19	17.48	56.77	123.45	15.88	100.36	136.82	99.62	94.65
ITC0653	23.88	1.689	10.71	503.26	151.35	46.14	327.06	745.83	31.53	56.59	202.00	21.64	93.45	130.81	98.83	92.58
ITC0653	23.45	1.105	6.242	607.14	50.18	72.87	245.30	683.32	21.19	29.93	134.65	27.81	97.53	148.17	104.47	95.19
ITC0653	23.09	1.947	6.181	769.81	115.96	53.58	432.71	687.51	33.18	35.86	189.06	62.12	95.39	170.30	102.95	94.04
ITC0654	22.51	1.198	4.415	623.67	186.71	50.02	248.37	654.19	29.05	88.38	121.44	46.62	99.29	162.59	102.88	94.60
ITC0654	21.90	2.315	8.264	868.42	185.59	73.09	450.41	869.13	61.95	81.61	216.10	91.85	101.92	149.94	104.29	95.80
ITC0654	22.93	1.689	4.922	472.68	333.14	68.12	291.53	586.62	44.86	42.87	112.95	31.13	101.06	174.13	109.13	95.47
ITC0659	22.82	1.815	5.269	371.82	33.55	64.89	234.63	375.45	14.07	31.27	128.87	64.29	96.77	142.04	97.58	93.99
ITC0659	24.64	1.891	8.198	351.61	41.36	46.50	253.61	376.03	22.41	43.35	149.10	77.52	96.63	198.90	107.62	94.44
ITC0659	24.02	1.114	11.08	318.06	109.36	63.21	213.21	374.43	24.61	58.34	122.87	86.88	94.13	184.48	103.46	94.19
ITC0662	21.68	0.620	2.971	88.59	34.34	66.55	141.14	245.15	23.65	17.16	79.28	59.39	101.07	153.23	103.03	95.10
ITC0662	27.15	1.994	29.16	857.99	156.63	51.12	377.04	483.99	25.25	35.53	147.36	28.12	98.62	127.10	104.32	94.92
ITC0662	26.76	1.502	2.831	709.42	136.25	61.23	306.07	456.83	20.77	23.22	146.14	41.43	103.80	139.70	107.10	96.46
ITC0767	30.92	1.593	4.517	140.64	218.15	63.05	15.43	452.42	25.04	33.88	114.30	41.80	101.81	178.50	111.53	102.76
ITC0767	25.58	0.628	6.661	103.85	44.81	53.17	12.89	831.66	16.89	51.67	169.00	36.36	101.87	168.69	108.74	97.12
ITC0767	28.85	1.709	6.064	78.93	141.93	66.72	22.80	678.41	35.18	31.40	154.30	56.44	97.28	196.42	111.77	93.99
ITC0767	26.49	1.686	5.754	128.30	202.91	63.26	21.33	633.23	33.15	33.38	146.31	53.52	103.69	209.83	108.77	99.32
ITC0767	28.15	1.367	2.923	214.17	46.85	72.92	11.61	600.29	20.13	62.14	156.97	29.84	101.00	218.06	117.84	97.56
ITC0767	29.64	1.142	6.515	184.10	40.78	61.38	13.96	518.87	17.47	63.04	142.22	35.59	99.77	231.04	116.02	97.54
ITC0769	24.42	1.437	7.052	299.32	30.25	56.77	39.27	457.82	27.11	44.06	132.96	18.58	99.57	177.22	104.25	94.31
ITC0769	22.83	2.161	11.23	317.55	14.46	71.74	73.12	495.79	28.71	78.62	172.96	40.23	98.59	168.67	101.00	94.25
ITC0769	25.44	1.529	5.735	406.13	17.56	17.73	43.70	470.14	14.46	84.43	146.81	20.34	96.51	168.33	100.84	92.91
ITC1120	27.75	1.517	9.158	437.05	20.45	77.52	43.61	811.07	20.79	25.61	238.81	37.50	91.09	137.45	93.69	86.35
ITC1120	34.09	1.578	27.07	546.83	28.76	104.12	55.04	682.54	31.28	28.61	158.94	54.95	95.43	143.36	98.29	90.01
ITC1120	24.08	1.138	17.76	504.73	22.90	45.47	108.78	519.86	32.59	25.12	118.81	44.17	95.54	191.62	98.98	92.75
ITC1121	20.33	0.694	3.450	239.98	14.34	46.94	227.90	165.83	20.75	17.46	65.74	19.71	98.83	208.07	100.74	94.36
ITC1121	21.53	0.621	3.729	357.05	152.02	50.35	252.28	197.14	20.71	25.68	66.82	28.53	105.51	216.91	107.04	100.46
ITC1121	21.31	0.904	3.479	520.88	27.24	47.15	476.04	340.83	32.31	26.85	155.60	32.81	102.88	176.33	103.01	94.91
ITC1122	22.40	1.673	5.603	1070.47	134.01	66.55	420.13	857.98	56.44	67.59	197.60	41.93	108.56	142.66	103.21	98.33
ITC1122	22.45	0.659	3.905	1140.82	135.90	57.58	305.64	683.19	37.08	36.70	168.56	41.47	97.50	160.19	106.07	91.39
ITC1122	21.48	1.926	6.027	788.91	126.42	48.14	448.74	901.66	61.27	75.31	204.85	42.04	102.32	200.04	107.21	95.59
ITC1177	22.31	0.641	5.457	112.59	169.82	70.06	24.48	569.03	19.17	42.04	100.22	75.14	101.77	188.21	104.60	94.79
ITC1177	22.00	0.878	6.762	106.86	216.74	70.19	30.64	692.26	23.56	40.07	118.77	95.94	101.34	185.72	106.38	95.16
ITC1177	26.42	0.713	5.421	231.22	247.00	56.14	32.52	637.66	25.92	63.97	137.03	78.81	97.04	178.68	115.70	96.88
ITC1177	23.23	1.473	9.786	257.16	108.96	63.70	45.64	563.00	21.60	43.24	132.28	28.89	96.88	179.46	111.27	97.70
ITC1177	20.64	0.886	9.191	259.68	133.47	45.49	66.53	457.96	32.52	41.59	78.88	24.85	104.68	195.20	104.32	95.29
ITC1177	26.42	1.089	9.246	188.43	103.80	49.11	36.88	464.96	17.13	31.88	102.29	24.15	103.22	195.67	115.43	94.96
ITC1177	22.01	1.413	6.282	245.75	291.24	64.46	33.05	555.84	21.33	65.95	130.37	112.89	100.65	175.42	102.55	94.19
ITC1187	26.48	0.959	18.61	967.13	98.79	85.96	52.63	1078.25	32.39	21.42	212.39	56.15	93.35	246.09	104.23	89.09
ITC1187	24.83	1.909	24.50	1273.34	159.35	80.87	57.75	1397.57	44.49	39.86	265.65	191.93	91.81	225.56	102.45	88.46
ITC1187	35.27	1.568	22.39	1163.54	137.63	66.69	55.91	1283.07	40.16	33.25	246.55	143.24	96.71	232.91	112.50	91.64
ITC1325	33.86	2.078	2.464	588.87	374.93	56.67	78.19	1271.71	65.64	56.78	270.88	117.79	113.28	264.47	113.93	106.74
ITC1325	29.27	1.888	4.392	631.72	25.42	52.28	34.79	788.76	24.72	122.44	159.29	55.54	100.98	179.32	103.55	95.00
ITC1325	23.66	1.556	4.374	724.87	44.89	14.19	58.79	908.28	31.34	219.15	199.51	42.14	98.99	144.25	99.75	93.93
ITC1441	21.23	0.913	2.833	187.37	215.31	45.00	17.61	361.93	16.70	54.38	97.04	17.43	88.64	153.57	94.48	85.02
ITC1441	22.53	1.562	5.952	446.27	151.68	47.57	47.26	947.57	19.93	28.40	285.65	24.52	99.46	161.47	100.54	92.94
ITC1441	23.26	1.032	3.621	318.05	25.69	72.29	29.11	590.59	21.11	21.49	158.85	13.13	98.83	146.92	97.12	93.25
ITC1441	24.79	1.108	5.878	393.79	71.00	66.92	36.37	802.01	19.24	16.03	223.73	34.99	112.14	193.55	103.17	99.73
ITC1441	24.69	1.138	5.583	215.29	61.09	57.94	37.21	551.93	31.11	38.45	186.69	24.12	99.86	184.67	101.41	94.19
ITC1441	20.55	0.956	4.556	266.44	205.57	43.54	18.47	521.44	18.94	57.21	140.80	21.85	96.70	157.47	93.81	88.25
ITC1441	26.91	1.704	5.808	345.22	242.72	54.66	19.94	612.78	24.82	38.27	155.15	16.88	97.71	191.56	102.64	93.20
ITC1483	40.51	1.483	6.064	176.15	88.28	80.21	22.64	475.72	36.71	188.65	120.56	48.83	108.04	280.40	112.36	103.58
ITC1483	35.93	1.903	6.072	160.49	223.88	65.37	16.14	477.16	38.85	122.71	102.47	29.81	103.60	313.31	110.22	98.36
ITC1483	36.68	1.171	4.664	186.82	482.28	50.14	12.51	632.21	18.36	61.05	148.07	24.49	100.89	227.16	109.17	94.89
ITC1483	29.52	1.435	3.063	266.05	67.21	59.20	13.95	681.52	22.18	87.34	163.09	24.02	108.96	238.51	109.41	96.41
ITC1483	30.39	1.957	5.565	250.11	282.50	52.46	21.43	834.41	34.35	30.26	194.97	46.87	92.76	256.29	106.02	89.74
ITC1483	33.05	1.422	4.990	127.15	133.00	50.10	12.70	616.57	30.07	53.91	140.54	46.15	96.69	203.05	111.52	92.39
ITC1483	32.54	1.847	15.20	343.65	106.80	49.61	21.08	958.09	52.19	165.89	211.01	45.69	97.71	271.68	112.65	94.29

Supplementary Figure S4. Pathway displays showing ratio difference between wild and cultivated species with the same genetic background.

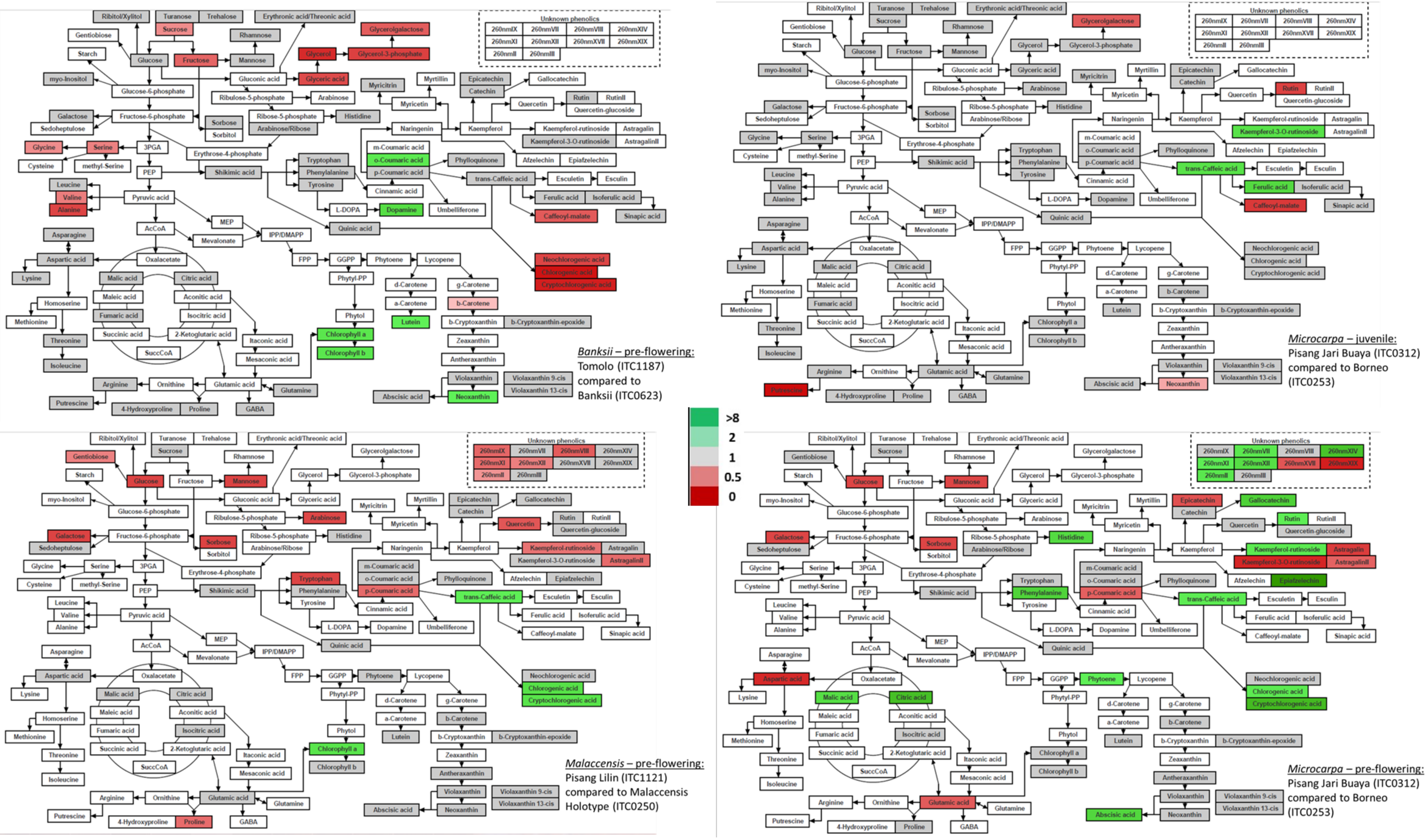

Supplementary Table S5. Differential expression of metabolites detected in juvenile leaves.

Juvenile leaves: difference between A (all varieties with only A genome) and B (varieties containing at least one B genome)

Features	p-value	Significant	Mean		Highest mean
			A	B	
Catechin	**0.000**	**Yes**	53.091	187.341	B
Caffeoyl-malate	**0.000**	**Yes**	227.805	37.817	A
Kaempferol-3-O-rutinoside	**0.000**	**Yes**	166.015	433.331	B
Asparagine	**0.000**	**Yes**	3030.824	5609.076	B
Epicatechin	**0.000**	**Yes**	1147.030	3569.931	B
Lutein	**0.000**	**Yes**	1.994	1.538	A
Pheophytin a	**0.000**	**Yes**	8.770	5.879	A
Rutin	**0.000**	**Yes**	115.106	62.465	A
Chlorophyll b	**0.000**	**Yes**	7.717	4.991	A
Neoxanthin	**0.000**	**Yes**	0.500	0.438	A
Cryptochlorogenic acid	**0.000**	**Yes**	4453.218	13771.922	B
Chlorophyll a	**0.000**	**Yes**	9.075	4.917	A
Violaxanthin	**0.000**	**Yes**	0.334	0.314	A
Neochlorogenic acid	**0.000**	**Yes**	1706.402	3860.136	B
Glutamic acid	**0.000**	**Yes**	197.883	318.103	B
Dopamine	**0.000**	**Yes**	22215.532	11717.685	A
alpha-methyl-DL-Serine/Homoserine/Threonine	**0.001**	**Yes**	1172.923	828.105	A
Violaxanthin isomer	**0.001**	**Yes**	0.334	0.314	A
Arginine	**0.001**	**Yes**	1634.977	3017.198	B
Violaxanthin 9-cis	**0.003**	**Yes**	0.348	0.327	A
Tryptophan	**0.003**	**Yes**	5090.495	3586.646	A
Phenylalanine	**0.005**	**Yes**	2933.199	2091.786	A
Quinic acid	**0.006**	**Yes**	24652.032	34639.048	B
Violaxanthin 13-cis	**0.008**	**Yes**	0.497	0.469	A
Isoleucine	**0.010**	**Yes**	109.123	71.559	A
Tyrosine	**0.015**	**Yes**	408.910	305.362	A
Ribitol/Xylitol	**0.028**	**Yes**	145.136	122.304	A
Abscisic acid	**0.028**	**Yes**	150.030	186.782	B
Serine	**0.037**	**Yes**	1919.865	2485.314	B
Leucine	**0.044**	**Yes**	141.093	95.798	A
Glycerolgalactose	**0.046**	**Yes**	2567.888	1771.453	A
Threonine	0.053	No	1037.216	830.638	
b-Cryptoxanthin-5,6-epoxide	0.057	No	0.322	0.311	
Proline	0.061	No	102.205	165.585	
Putrescine	0.069	No	93.374	0.000	
p-Coumaric acid	0.108	No	421.825	325.246	
Glycerol-3-P	0.133	No	214.937	269.220	
4-Hydroxyproline	0.281	No	82.750	90.810	
Glucose/Galactose	0.401	No	11837.810	19342.630	
Chlorogenic acid	0.610	No	2770.166	1825.880	
Turanose/Trehalose	0.725	No	3955.942	3590.903	
Aspartic acid	0.824	No	2140.361	2358.003	
Mannose	0.959	No	41267.067	1029.313	
Ferulic acid	0.992	No	200.449	164.952	
bCarotene	1.000	No	0.859	0.847	
o-Coumaric acid	1.000	No	96.993	78.347	
Fructose	1.000	No	23312.526	28202.938	
Myricitrin	1.000	No	142.670	129.979	
Shikimic acid	1.000	No	1825.810	1980.616	
Rhamnose	1.000	No	4700.466	4937.883	
Citric acid	1.000	No	5656.121	4918.947	
Citric acid (GC)	1.000	No	92.871	71.996	
Malic acid (GC)	1.000	No	1417.660	1553.042	
Phenylalanine (GC)	1.000	No	70.514	49.987	
Malic acid	1.000	No	12190.063	13135.119	
Isoferulic acid	1.000	No	560.792	511.159	
GABA	1.000	No	1568.982	1903.662	
Glucose/Mannose/Galactose/Sorbose	1.000	No	10648.840	11227.394	
Turanose (GC)	1.000	No	283.180	337.125	
Methylphosphate	1.000	No	22.610	26.497	
Glycerol	1.000	No	607.866	687.456	
Glycine	1.000	No	351.701	413.844	
Phylloquinone	1.000	No	0.057	0.057	
Pyroglutamic acid	1.000	No	5869.863	5995.452	
myo-Inositol (GC)	1.000	No	1870.345	1842.894	
Aspartic acid (GC)	1.000	No	222.646	272.271	
Phosphate	1.000	No	6604.330	6321.633	
Dopamine (GC)	1.000	No	0.000	0.000	
Valine	1.000	No	332.940	324.235	
Glyceric acid	1.000	No	73.086	71.716	
Fumaric acid	1.000	No	0.000	0.000	
Quinone	1.000	No	0.035	0.034	
Arabinose/Ribose	1.000	No	2430.632	2579.782	
Histidine	1.000	No	1651.488	1598.741	
Erythronic acid/Threonic acid	1.000	No	137.384	137.394	
myo-Inositol	1.000	No	2051.539	2303.516	
Caffeic acid	1.000	No	803.499	860.238	
Glutamine	1.000	No	110.055	101.181	
Sinapic acid	1.000	No	105.893	114.293	
Lysine	1.000	No	35649.205	36127.891	
Disaccharide 47.6min	1.000	No	83.816	86.583	
Sucrose	1.000	No	75149.746	77769.667	
Oxalic acid	1.000	No	140.302	163.913	
Glucose	1.000	No	62809.490	61013.599	

Juvenile leaves: difference between genotypes AAw, Aacv, AAA, AAB, AB, ABB, BB and Rhodoclamys

Features	p-value	Significant	Mean								Highest mean	Lowest mean
			AAA	AAB	AB	AAw	AAcv	ABB	Rhodoclamys	BB		
Caffeoyl-malate	**0.000**	**Yes**	123.1	37.8	154.7	297.5	242.4	43.9	27.0	24.5	AAw	BB
Catechin	**0.000**	**Yes**	40.2	137.6	204.9	50.9	67.4	165.4	129.8	372.9	BB	AAA
Chlorogenic acid	**0.000**	**Yes**	675.0	1288.0	479.9	5497.1	1156.8	1245.8	2975.3	5183.6	AAw	AB
Cryptochlorogenic acid	**0.000**	**Yes**	8240.1	16884.5	5163.9	3842.8	4517.4	7081.1	29067.1	22668.8	Rhodoclamys	AAw
Asparagine	**0.000**	**Yes**	3721.1	4389.3	5213.6	2866.9	3676.0	5423.0	1978.3	13844.2	BB	Rhodoclamys
Rutin	**0.000**	**Yes**	92.2	48.9	90.3	146.3	72.8	66.2	764.0	67.6	Rhodoclamys	AAB
alpha-methyl-DL-Serine/Homoserine/Threonine	**0.000**	**Yes**	1580.8	1172.6	855.8	1067.0	1454.2	674.2	530.2	767.8	AAA	Rhodoclamys
Glycerolgalactose	**0.000**	**Yes**	4208.9	2601.2	1008.7	2687.3	1181.2	2040.6	1310.5	977.3	AAA	BB
Kaempferol-3-O-rutinoside	**0.000**	**Yes**	131.6	426.7	274.6	189.8	158.3	399.7	129.3	591.4	BB	Rhodoclamys
Neochlorogenic acid	**0.000**	**Yes**	2993.9	2781.9	2886.8	1265.0	3462.1	4078.2	6657.8	7845.1	BB	AAw
Pheophytin a	**0.000**	**Yes**	9.5	7.1	4.0	8.9	8.7	4.7	5.5	5.4	AAA	AB
myo-Inositol (GC)	**0.000**	**Yes**	1482.1	2503.8	966.1	1951.7	1910.0	1221.2	3802.8	1529.0	Rhodoclamys	AB
Arginine	**0.000**	**Yes**	2213.5	1593.9	5424.8	1590.1	2151.3	4038.6	234.9	2420.4	AB	Rhodoclamys
Lutein	**0.000**	**Yes**	2.1	1.7	1.0	2.0	1.9	1.2	1.5	1.6	AAA	AB
Phosphate	**0.000**	**Yes**	3283.0	9227.7	6114.1	7852.8	5018.6	5413.7	9077.1	7810.2	AAB	AAA
Epicatechin	**0.000**	**Yes**	734.3	3569.9	2624.5	1898.8	1187.5	3327.7	947.1	5453.5	BB	AAA
Chlorophyll b	**0.000**	**Yes**	8.4	6.1	3.3	8.3	7.2	4.4	4.6	5.4	AAA	AB
Threonine	**0.000**	**Yes**	1314.1	848.2	1007.5	879.1	1325.1	762.3	527.9	484.3	AAcv	BB
Neoxanthin	**0.000**	**Yes**	0.5	0.5	0.3	0.5	0.5	0.4	0.4	0.4	AAA	AB
Dopamine	**0.000**	**Yes**	17501.6	6984.5	12430.6	24283.9	29301.0	14809.0	8620.8	23640.2	AAcv	AAB
Disaccharide 47.6min	**0.000**	**Yes**	198.7	0.0	128.4	53.3	144.6	270.4	118.9	103.0	ABB	AAB
Violaxanthin	**0.000**	**Yes**	0.3	0.3	0.3	0.3	0.3	0.3	0.3	0.3	AAw	AB
Phenylalanine	**0.000**	**Yes**	2926.5	2151.9	2949.9	3352.3	2936.3	1604.5	1022.6	2080.4	AAw	Rhodoclamys
Methylphosphate	**0.000**	**Yes**	14.2	34.2	22.5	35.0	20.2	16.0	21.1	10.9	AAw	BB
Ferulic acid	**0.000**	**Yes**	282.6	101.5	271.1	177.7	169.3	114.6	86.3	244.2	AAA	Rhodoclamys
Chlorophyll a	**0.000**	**Yes**	9.7	6.4	2.2	9.0	9.2	4.3	4.5	6.1	AAA	AB
o-Coumaric acid	**0.000**	**Yes**	70.8	132.4	99.6	97.4	143.7	50.9	52.7	61.6	AAcv	ABB
Quinic acid	**0.000**	**Yes**	24652.0	37482.8	37579.6	21805.1	26602.0	27361.8	48606.0	30545.8	Rhodoclamys	AAw
bCarotene	**0.000**	**Yes**	0.9	0.9	0.8	0.9	0.8	0.8	0.9	0.9	AAB	ABB
p-Coumaric acid	**0.000**	**Yes**	413.8	280.6	492.4	432.0	471.6	286.2	233.2	509.2	BB	Rhodoclamys
Leucine	**0.000**	**Yes**	147.8	127.1	111.4	147.9	132.2	69.7	0.0	23.2	AAw	Rhodoclamys
Violaxanthin isomer	**0.000**	**Yes**	0.3	0.3	0.3	0.3	0.3	0.3	0.3	0.3	AAA	AB
Aspartic acid	**0.001**	**Yes**	2631.0	2053.8	2544.8	2015.7	2459.0	2066.8	1716.0	3018.0	BB	Rhodoclamys
Sinapic acid	**0.001**	**Yes**	79.5	103.5	160.7	119.9	250.1	69.5	34.4	209.3	AAcv	Rhodoclamys
myo-Inositol	**0.001**	**Yes**	3475.4	2898.9	1817.0	1886.3	2194.1	2308.7	2089.3	1691.6	AAA	BB
Fructose	**0.001**	**Yes**	17929.4	35771.5	21276.8	25660.6	13549.6	20225.8	37456.0	20534.0	Rhodoclamys	AAcv
Glutamic acid	**0.001**	**Yes**	232.5	372.9	255.1	174.4	228.3	257.9	181.2	428.3	BB	AAw
Histidine	**0.001**	**Yes**	1697.6	1803.0	2484.0	1592.0	1918.8	1190.3	911.2	1297.3	AB	Rhodoclamys
Abscisic acid	**0.001**	**Yes**	173.4	177.0	112.7	138.4	132.5	188.6	161.6	217.7	BB	AB
Glyceric acid	**0.001**	**Yes**	44.5	84.7	49.9	86.8	56.2	49.9	130.9	84.6	Rhodoclamys	AAA
Isoleucine	**0.001**	**Yes**	128.5	81.0	90.3	108.5	112.9	46.4	40.2	0.0	AAA	BB
Violaxanthin 9-cis	**0.001**	**Yes**	0.3	0.3	0.3	0.4	0.3	0.3	0.3	0.3	AAw	BB
Glucose/Galactose	**0.001**	**Yes**	22140.4	17042.4	13922.9	10117.8	9195.4	35111.1	11358.5	17568.3	ABB	AAcv
Rhamnose	**0.001**	**Yes**	4700.5	6206.7	3246.7	5252.4	4145.9	4494.9	1549.2	3701.2	AAB	Rhodoclamys
Serine	**0.001**	**Yes**	2014.7	2980.3	2697.2	1942.2	2157.1	2269.7	928.6	1644.2	AAB	Rhodoclamys
GABA	**0.001**	**Yes**	1331.8	2160.1	1747.8	1557.7	1758.8	1204.2	995.3	823.2	AAB	BB
Tyrosine	**0.002**	**Yes**	496.8	304.5	424.7	383.3	439.6	289.0	269.0	237.1	AAA	BB
Violaxanthin 13-cis	**0.002**	**Yes**	0.5	0.5	0.4	0.5	0.5	0.4	0.5	0.5	AAcv	AB
Valine	**0.002**	**Yes**	371.2	415.9	295.2	334.8	342.8	251.2	178.7	127.9	AAB	BB
Glycerol-3-P	**0.002**	**Yes**	194.3	474.9	193.6	232.0	153.9	163.6	232.1	318.6	AAB	AAcv
b-Cryptoxanthin-5,6-epoxide	**0.004**	**Yes**	0.3	0.3	0.3	0.3	0.3	0.3	0.3	0.3	AAB	AB
Isoferulic acid	**0.004**	**Yes**	690.0	322.4	522.7	520.8	775.3	406.1	316.4	830.8	BB	Rhodoclamys
Lysine	**0.005**	**Yes**	43559.2	44723.4	35250.7	32554.7	39561.7	31986.5	39154.2	29835.7	AAB	BB
Myricitrin	**0.006**	**Yes**	152.4	143.7	129.2	132.6	157.3	110.7	3473.2	123.9	Rhodoclamys	ABB
Putrescine	**0.007**	**Yes**	138.2	0.0	76.2	115.9	17.5	0.0	0.0	30.3	AAA	AAB
Caffeic acid	**0.007**	**Yes**	1083.4	627.4	838.0	643.2	951.9	632.7	928.9	1628.0	BB	AAB
Glucose/Mannose/Galactose/Sorbose	**0.009**	**Yes**	15571.5	15727.6	8993.6	9143.9	10527.1	11013.9	10120.1	9468.0	AAB	AB
Glycine	**0.009**	**Yes**	296.3	462.3	287.1	355.5	428.9	286.3	376.7	208.1	AAB	BB
Ribitol/Xylitol	**0.012**	**Yes**	166.4	127.4	94.4	128.3	143.1	107.4	138.7	123.3	AAA	AB
Quinone	**0.016**	**Yes**	0.0	0.0	0.0	0.0	0.0	0.0	0.0	0.0	Rhodoclamys	AAB
Erythronic acid/Threonic acid	**0.027**	**Yes**	80.0	183.3	113.4	158.4	183.5	123.1	129.8	108.7	AAcv	AAA
Tryptophan	**0.033**	**Yes**	5078.1	3698.7	3897.7	5633.7	4398.7	3482.6	6651.9	3321.1	Rhodoclamys	BB
4-Hydroxyproline	**0.045**	**Yes**	90.6	96.8	72.9	76.8	96.1	82.8	84.0	103.2	BB	AB
Shikimic acid	0.075	No	2186.4	1971.6	2377.0	1390.9	1890.6	1773.7	2862.8	2206.6		
Glucose	0.166	No	81406.7	53984.9	55139.6	54758.8	51021.5	89045.1	76383.4	52700.7		
Turanose/Trehalose	0.188	No	5152.6	3572.4	3582.5	3524.4	4667.2	3725.3	3951.5	3730.1		
Glycerol	0.262	No	607.9	888.7	505.8	678.0	473.2	811.5	281.9	477.8		
Phylloquinone	0.268	No	0.1	0.1	0.1	0.1	0.1	0.1	0.0	0.1		
Turanose (GC)	0.320	No	141.3	431.2	53.7	283.0	332.6	0.0	0.0	0.0		
Mannose	0.457	No	43980.7	0.0	57115.2	42375.9	10113.2	0.0	65961.3	22593.7		
Proline	0.461	No	102.2	185.9	159.2	108.2	105.6	185.7	90.0	110.9		
Malic acid	0.461	No	12467.1	12871.7	12571.5	11506.8	15831.2	13876.3	13435.0	14625.0		
Citric acid	0.589	No	5405.8	5048.7	4838.8	5366.4	6344.1	5329.0	8846.5	5172.0		
Pyroglutamic acid	0.638	No	5869.9	8244.7	5536.4	5545.9	5955.7	5858.1	10741.1	4910.1		
Glutamine	0.683	No	148.2	13.5	101.4	62.5	107.0	317.6	154.2	101.5		
Arabinose/Ribose	0.684	No	2491.0	2567.4	1867.2	2018.3	2971.4	2825.0	3993.7	2840.7		
Oxalic acid	0.731	No	111.9	153.9	64.8	156.9	130.9	275.1	235.7	226.6		
Phenylalanine (GC)	0.942	No	40.9	72.0	41.6	71.6	76.4	79.5	0.0	15.9		
Citric acid (GC)	1.000	No	97.2	0.0	81.1	82.9	142.1	110.9	73.5	96.7		
Malic acid (GC)	1.000	No	1264.2	1505.0	1310.3	1387.4	1590.6	1964.2	1926.9	1300.2		
Aspartic acid (GC)	1.000	No	170.2	266.6	235.3	255.6	266.9	571.3	239.3	241.7		
Sucrose	1.000	No	70373.0	72095.3	69808.9	75393.8	75743.9	87461.7	88849.4	72153.1		
Dopamine (GC)	1.000	No	0.0	6360.4	0.0	0.0	0.0	0.0	7978.6	0.0		
Fumaric acid	1.000	No	0.0	0.0	21.8	0.0	6.3	0.0	11.3	0.0		

Supplementary Table S6. Differential expression of metabolites detected in pre-flowering leaves.

Pre-flowering leaves: difference between A (all varieties with only A genome) and B (varieties containing at least one B genome)

Features	p-value	Significant	Mean A	Mean B	Highest mean
Epiafzelechin	**0.000**	**Yes**	18.81	516.59	B
Rutin	**0.000**	**Yes**	236.60	24.70	A
260nm(VII)	**0.000**	**Yes**	104.98	300.40	B
Kaempferol-rutinoside	**0.000**	**Yes**	319.21	557.70	B
260nm(VIII)	**0.000**	**Yes**	18.90	51.88	B
kaempferol-3-O-rutinoside	**0.000**	**Yes**	52.56	13.11	A
Quercetin-glucoside	**0.000**	**Yes**	657.55	318.06	A
260nm(XIV)	**0.000**	**Yes**	208.89	115.97	A
Quinic acid	**0.001**	**Yes**	157.99	87.39	A
260nm(XVII)	**0.001**	**Yes**	219.24	142.40	A
Phytoene	**0.001**	**Yes**	22.53	25.44	B
Citric acid	**0.001**	**Yes**	3112.14	1666.73	A
260nm(XIII)	**0.002**	**Yes**	9.72	17.58	B
260nm(XI)	**0.003**	**Yes**	225.61	169.58	A
260nm(XII)	**0.006**	**Yes**	135.11	183.12	B
Pheophytin a	**0.008**	**Yes**	820.05	525.57	A
Catechin	**0.020**	**Yes**	7.41	10.08	B
Tryptophan	**0.033**	**Yes**	51.32	37.42	A
260nm(IX)	0.052	No	35.49	24.09	
Isocitric acid	0.089	No	1274.36	833.05	
Quercetin	0.098	No	7.46	5.58	
o-Coumaric acid	0.119	No	5.70	4.18	
Glucuronic acid	0.224	No	14.35	12.52	
Cryptochlorogenic acid	0.338	No	2174.47	1850.33	
Chlorophyll b'	0.356	No	151.47	136.62	
Histidine	0.438	No	2.87	2.29	
AstragalinII	0.500	No	25.36	30.63	
Astragalin	0.500	No	207.66	135.61	
Epicatechin	0.582	No	488.29	610.96	
RutinII	0.656	No	559.42	612.78	
Sedoheptulose	0.699	No	29.98	26.82	
Sucrose	0.785	No	135.84	154.30	
Malic acid	0.889	No	842.31	679.57	
Violaxanthin	1.000	No	177.50	181.57	
Neoxanthin	1.000	No	185.35	195.30	
260nm(III)	1.000	No	682.56	453.14	
Phenylalanine	1.000	No	41.62	38.33	
Gentiobiose	1.000	No	64.81	72.85	
Aspartic acid	1.000	No	18.72	20.87	
Violaxanthin	1.000	No	97.63	99.11	
Proline	1.000	No	1.32	1.42	
Gallocatechin	1.000	No	5.20	5.41	
Chlorophyll b	1.000	No	1861.90	1585.59	
Chlorophyll a'	1.000	No	322.58	308.63	
Chlorophyll a	1.000	No	4589.08	4802.42	
Glucose/Mannose/Galactose/Sorbose	1.000	No	60.01	50.72	
Violaxanthin 9-cis	1.000	No	94.32	94.31	
Quinone	1.000	No	56.86	55.31	
b-Cryptoxanthin-5,6-epoxide	1.000	No	92.70	92.68	
Caffeic acid	1.000	No	25.13	27.54	
p-Coumaric acid	1.000	No	13.59	11.79	
Chlorogenic acid	1.000	No	59.32	58.06	
260nm(XIX)	1.000	No	36.31	44.80	
b-Carotene	1.000	No	277.23	277.50	
Inositol-myo	1.000	No	120.24	110.58	
Arabinose/Ribose	1.000	No	59.23	51.27	
Glutamic acid	1.000	No	41.30	35.52	
Neochlorogenic acid	1.000	No	663.63	736.33	
Abscisic acid	1.000	No	4.56	4.76	
Phylloquinone	1.000	No	20.67	20.49	
Guanine	1.000	No	2.90	2.56	
Lutein	1.000	No	575.04	569.44	
Antheraxanthin	1.000	No	105.00	105.12	
m-Coumaric acid	1.000	No	5.94	5.81	
Shikimic acid	1.000	No	43.05	49.96	
Violaxanthin 13-cis	1.000	No	104.25	103.55	

Pre-flowering leaves: difference between genotypes AAw, Aacv, AAA, AAB, AB, ABB and BB

Features	p-value	Significant	Mean AAA	AAB	ABB	AB	AAw	AAcv	BB	Highest mean	Lowest mean
Epiafzelechin	**0.000**	**Yes**	18.17	337.04	1675.53	197.34	19.37	18.84	24.11	ABB	AAA
Rutin	**0.000**	**Yes**	148.39	31.95	17.37	32.92	427.50	193.27	55.04	AAw	ABB
Quercetin-glucoside	**0.000**	**Yes**	761.53	407.73	184.10	328.01	435.54	608.68	504.73	AAA	ABB
260nm(XIV)	**0.000**	**Yes**	209.22	173.00	73.57	79.43	173.05	336.87	121.83	AAcv	ABB
Kaempferol-rutinoside	**0.000**	**Yes**	391.04	584.61	584.61	199.95	243.04	379.88	807.64	BB	AB
260nm(VII)	**0.000**	**Yes**	160.40	313.33	263.56	340.07	73.06	133.22	397.44	BB	AAw
260nm(VIII)	**0.000**	**Yes**	30.21	52.89	51.74	31.09	21.43	12.27	65.40	BB	AAcv
Kaempferol-3-O-rutinoside	**0.001**	**Yes**	42.29	15.85	11.91	5.95	243.08	44.05	34.62	AAw	AB
260nm(XII)	**0.001**	**Yes**	158.74	179.18	184.36	70.69	109.45	168.79	227.44	BB	AB
260nm(XIII)	**0.001**	**Yes**	9.45	12.19	37.57	8.58	9.88	12.19	71.10	BB	AB
Catechin	**0.001**	**Yes**	5.07	8.50	16.70	4.57	8.30	7.57	10.79	ABB	AB
Cryptochlorogenic acid	**0.002**	**Yes**	3717.06	1999.14	1801.60	1194.08	899.14	4407.50	336.40	AAcv	BB
Quinic acid	**0.002**	**Yes**	259.92	61.53	109.36	105.43	151.65	144.49	22.90	AAA	BB
Neoxanthin	**0.002**	**Yes**	173.27	184.09	235.14	158.67	201.90	182.90	145.67	ABB	BB
260nm(XVII)	**0.002**	**Yes**	196.41	144.43	135.65	48.96	268.94	184.06	228.10	AAw	AB
Phytoene	**0.003**	**Yes**	22.42	24.89	28.85	29.10	22.40	24.35	27.75	AB	AAw
260nm(XIX)	**0.003**	**Yes**	52.53	57.52	21.69	20.59	31.91	42.06	12.41	AAB	BB
RutinII	**0.005**	**Yes**	692.76	781.46	591.62	418.95	387.35	605.01	682.54	AAB	AAw
Citric acid	**0.006**	**Yes**	3994.90	2131.65	1461.99	885.70	3423.66	1414.38	1144.50	AAA	AB
Pheophytin a	**0.006**	**Yes**	589.19	513.15	579.44	785.80	1160.86	819.68	471.10	AAw	BB
260nm(IX)	**0.012**	**Yes**	25.63	20.47	33.40	3.38	35.49	47.34	7.70	AAcv	AB
Epicatechin	**0.012**	**Yes**	498.04	583.04	690.77	263.32	626.45	387.17	707.79	BB	AB
260nm(XI)	**0.013**	**Yes**	234.15	167.05	169.58	136.96	214.83	250.45	281.10	BB	AB
AstragalinII	**0.016**	**Yes**	27.44	42.50	24.19	38.82	18.14	33.12	65.82	BB	AAw
Quercetin	**0.016**	**Yes**	6.06	5.40	5.75	16.94	9.08	6.21	17.76	BB	AAB
Chlorophyll b	**0.019**	**Yes**	1772.41	1481.68	2058.03	950.17	1935.84	1752.32	901.61	ABB	BB
Astragalin	**0.019**	**Yes**	189.82	235.91	96.20	76.64	270.12	213.03	132.15	AAw	AB
Violaxanthin isomer2	**0.019**	**Yes**	161.39	176.57	204.41	145.53	181.34	193.04	143.36	ABB	BB
Shikimic acid	**0.025**	**Yes**	78.46	57.00	46.70	34.01	46.96	31.59	25.61	AAA	BB
o-Coumaric acid	**0.028**	**Yes**	4.36	3.99	4.18	6.33	6.76	6.36	3.71	AAw	BB
Violaxanthin 13-cis	**0.028**	**Yes**	104.12	102.56	109.18	98.50	105.49	102.70	98.29	ABB	BB
Chlorophyll a	**0.030**	**Yes**	4499.33	4633.64	5765.99	1146.44	4094.65	5882.92	2206.35	AAcv	AB
Chlorogenic acid	**0.037**	**Yes**	103.38	61.45	53.12	40.72	27.48	79.12	75.67	AAA	AAw
p-Coumaric acid	**0.049**	**Yes**	11.08	9.94	16.63	14.67	17.33	14.37	45.33	BB	AAB
Tryptophan	0.079	No	41.98	25.84	41.71	40.67	70.98	59.13	44.17		
Lutein	0.079	No	560.08	501.68	679.14	540.35	601.97	569.06	412.22		
Isocitric acid	0.087	No	1493.84	925.47	839.89	462.03	1540.02	782.86	599.74		
Neochlorogenic acid	0.087	No	850.68	841.42	661.18	355.35	226.33	1226.14	1065.91		
Caffeic acid	0.087	No	31.59	24.91	28.96	24.60	19.07	29.94	31.40		
Histidine	0.128	No	2.82	2.40	2.33	2.04	2.53	4.43	1.93		
b-Cryptoxanthin-5,6-epoxide	0.128	No	92.83	92.79	93.84	89.85	93.21	90.62	89.49		
Chlorophyll b'	0.133	No	149.76	112.87	155.39	172.80	174.81	146.33	126.34		
Violaxanthin 9-cis	0.144	No	94.76	94.48	95.46	91.22	94.88	93.17	90.01		
Phenylalanine	0.149	No	43.88	30.25	42.87	26.53	35.98	52.12	35.43		
Antheraxanthin	0.154	No	107.02	104.49	106.62	105.06	105.87	103.16	103.57		
260nm(II)	0.231	No	21.49	23.75	24.00	20.21	19.38	26.13	36.84		
Chlorophyll a'	0.250	No	333.28	298.40	320.32	455.30	360.91	292.88	245.11		
Gallocatechin	0.250	No	4.95	5.03	5.93	4.50	4.24	5.80	7.20		
260nm(III)	0.276	No	694.64	558.81	427.21	214.08	761.02	368.39	142.38		
Glucuronic acid	0.296	No	22.20	11.00	14.49	11.71	13.59	12.48	12.95		
Sucrose	0.330	No	124.58	160.78	148.10	99.17	134.67	150.43	158.94		
Violaxanthin isomer1	0.347	No	99.36	99.28	100.89	95.23	98.21	96.91	95.43		
b-Carotene	0.398	No	272.80	274.67	279.67	428.07	281.51	265.75	214.10		
Quinone	0.399	No	49.08	53.81	58.69	66.93	62.59	60.13	77.52		
Guanine	0.580	No	3.79	3.07	1.69	2.19	2.89	2.40	3.27		
Gentiobiose	0.637	No	63.84	73.43	71.20	50.29	64.81	71.04	81.53		
Arabinose/Ribose	0.866	No	66.63	51.22	54.09	46.46	61.77	45.36	68.56		
Sedoheptulose	0.981	No	31.95	26.47	30.06	14.61	23.83	31.92	31.28		
Malic acid	0.988	No	1099.38	765.08	677.90	558.42	648.63	767.21	549.51		
Proline	1.000	No	1.46	1.41	1.48	0.93	1.32	1.21	1.52		
Glutamic acid	1.000	No	33.91	38.11	35.39	23.00	48.27	48.21	44.34		
Glucose/Mannose/Galactose/Sorbose	1.000	No	62.09	53.67	51.06	60.01	60.09	52.01	29.79		
Phylloquinone	1.000	No	19.13	20.14	20.83	21.32	20.88	21.14	18.44		
Abscisic acid	1.000	No	4.66	4.75	4.66	2.95	4.10	6.52	7.97		
m-Coumaric acid	1.000	No	5.30	5.26	6.02	8.58	7.31	6.03	5.81		
myo-Inositol	1.000	No	121.51	114.98	109.40	97.80	116.04	110.43	122.16		
